# ERECTA, salicylic acid, abscisic acid, and jasmonic acid modulate quantitative disease resistance of *Arabidopsis thaliana* to *Verticillium longisporum*

**DOI:** 10.1186/1471-2229-14-85

**Published:** 2014-04-01

**Authors:** Eva Häffner, Petr Karlovsky, Richard Splivallo, Anna Traczewska, Elke Diederichsen

**Affiliations:** 1 Freie Universität Berlin, Fachbereich Biologie, Chemie, Pharmazie, Institut für Biologie, Dahlem Centre of Plant Sciences, Angewandte Genetik, Albrecht-Thaer-Weg 6, 14195 Berlin, Germany; 2 Department of Crop Sciences, Georg-August-Universität Göttingen, Molecular Phytopathology and Mycotoxin Research Section, Grisebachstraße 6, 37077 Göttingen, Germany

**Keywords:** *Arabidopsis thaliana*, *Verticillium longisporum*, QDR, RIL, NIL, QTL, *Erecta*, ABA, JA, SA

## Abstract

**Background:**

*Verticillium longisporum* is a soil-borne vascular pathogen infecting cruciferous hosts such as oilseed rape. Quantitative disease resistance (QDR) is the major control means, but its molecular basis is poorly understood so far. Quantitative trait locus (QTL) mapping was performed using a new (Bur×L*er*) recombinant inbred line (RIL) population of *Arabidopsis thaliana*. Phytohormone measurements and analyses in defined mutants and near-isogenic lines (NILs) were used to identify genes and signalling pathways that underlie different resistance QTL.

**Results:**

QTL for resistance to *V. longisporum*-induced stunting, systemic colonization by the fungus and for *V. longisporum*-induced chlorosis were identified. Stunting resistance QTL were contributed by both parents. The strongest stunting resistance QTL was shown to be identical with *Erecta*. A functional *Erecta* pathway, which was present in Bur, conferred partial resistance to *V. longisporum*-induced stunting. Bur showed severe stunting susceptibility in winter. Three stunting resistance QTL of L*er* origin, two co-localising with wall-associated kinase-like (*Wakl*)*-*genes, were detected in winter. Furthermore, Bur showed a much stronger induction of salicylic acid (SA) by *V. longisporum* than L*er*. Systemic colonization was controlled independently of stunting. The *vec1* QTL on chromosome 2 had the strongest effect on systemic colonization. The same chromosomal region controlled the level of abscisic acid (ABA) and jasmonic acid (JA) in response to *V. longisporum*: The level of ABA was higher in colonization-susceptible L*er* than in colonization-resistant Bur after *V. longisporum* infection. JA was down-regulated in Bur after infection, but not in L*er*. These differences were also demonstrated in NILs, varying only in the region containing *vec1*. All phytohormone responses were shown to be independent of *Erecta*.

**Conclusions:**

Signalling systems with a hitherto unknown role in the QDR of *A. thaliana* against *V. longisporum* were identified: *Erecta* mediated resistance against *V. longisporum*-induced stunting. Independent of *Erecta*, stunting was caused in a light-dependent manner with possible participation of SA and *Wakl* genes. ABA and JA showed a genotype-specific response that corresponded with systemic colonization by the fungus. Understanding the biological basis of phenotypic variation in *A. thaliana* with respect to *V. longisporum* resistance will provide new approaches for implementing durable resistance in cruciferous crops.

## Background

Quantitative disease resistance (QDR) is a complex phenomenon involving a plethora of molecular mechanisms [1,2]. It is often a sustainable form of resistance relying on multiple genes and effects which cannot easily be overcome by the pathogen’s adaptation. *Verticillium longisporum* is a soil-borne vascular pathogen of recent evolutionary origin with a host range centred on crucifers [3]. QDR is the only form of resistance against this pathogen described so far. *V. longisporum* causes significant and increasing yield losses on oilseed rape [4-7]. The fungus enters the host via the root and, at the onset of flowering, spreads systemically inside the xylem, thereby colonizing the whole plant [5,8,9]. *V. longisporum* induces early senescence [6,10] and colonizes senescent tissue to form microsclerotia that persist in the soil [11]. Since the disease is difficult to control, durable resistance in host plants is highly desirable.

Quantitative resistance against *V. longisporum* has been described for various accessions of *Brassica* species [12-15] (Konietzki and Diederichsen unpublished) and also for *A. thaliana* ecotypes [8,10,16,17]. In some cases, QTL have been identified which control resistance traits, such as fresh weight [16], chlorosis [10,16], systemic colonization, stunting, and axillary branching [8], or the area under the disease progression curve in *Brassica* species [15] (Konietzki and Diederichsen unpublished). However, the underlying genes and regulatory processes have rarely been identified. Secondary metabolism plays a role in resistance: A resistant line of *B. napus* produced more phenolic substances in the xylem of the hypocotyl upon infection than a susceptible line [18]. Indeed, QTL for contents of phenylpropanoid compounds co-localised with resistance QTL in *B. napus*[19], and it was shown that soluble phenylpropanoids played a role in *A. thaliana* defence against *V. longisporum*[20]. *Rfo1* has been shown to mediate resistance against *V. longisporum*-induced fresh weight loss in *A. thaliana*[16], and encodes a wall-associated kinase-like (WAKL) -protein that conferred resistance against *Fusarium oxysporum*[21].

Recent research has revealed different processes that are involved in the host’s response to *V. longisporum* on the molecular level: Ethylene signalling plays either a protective or a deleterious role, depending on the signalling components involved [16,22]. *V. longisporum* infection caused elevated levels of salicylic acid (SA) in the xylem of *Brassica* shoots [23]. Previous studies suggest that jasmonic acid (JA) signalling does not play a role in the host-pathogen interaction [10,23], but the JA receptor COI1 promotes the disease in a JA-independent, yet unknown way [24]. Whereas an interplay of indole glucosinolates and camalexin has been shown to be involved in early defence against *V. longisporum* in *A. thaliana* roots [25], reactive oxygen species played a role in defence during the later stages of the disease [26]. Furthermore, the nuclear-localised *ahl19* gene acted as a positive regulator of defence to *V. longisporum* and other *Verticillium* species in *A. thaliana*[27]. Several apoplastic enzymes were induced by *V. longisporum* in *Brassica* and possibly play a role in defence [28]. The host’s reaction to the pathogen involves trans-differentiation of bundle sheath cells into functional xylem elements under the control of the vascular-related NAC domain 7 transcription factor [29].

However, little is known about how these processes relate to QDR. It is unclear whether resistance QTL represent genes within regulatory systems that have already been shown to operate in the host-pathogen interaction, or whether they constitute new components, adding to the complexity of the pathosystem. Furthermore, the role of known defence signalling pathways in natural resistance to *V. longisporum* is poorly understood so far.

The present study aims at identifying genes and signalling pathways that account for differences in QDR against *V. longisporum* in *A. thaliana*. QTL for relevant resistance traits using a new (Bur×L*er*) recombinant inbred line (RIL) population have been identified. It is shown that the *Erecta* gene corresponded to a strong QTL mediating stunting resistance and that a functional *Erecta* signalling pathway mediated resistance against *V. longisporum*-induced stunting. Evidence is provided that SA, abscisic acid (ABA) and JA contents responded to *V. longisporum* in a genotype-specific way, and that changes in ABA and JA content were controlled by the same QTL that also conferred resistance to systemic colonization by the fungus.

## Methods

### Material

*A. thaliana* ecotypes Bur-0, Col-0 and L*er*-0 were originally obtained from the Arabidopsis Information Service (AIS) Frankfurt [30] and maintained in-house. All other *A. thaliana* genotypes were obtained from the Nottingham Arabidopsis Stock Centre (NASC). *er-105*, *er-108, er-111*, and *er-118* were included as strong *erecta* mutants, whilst *er-116* represented a weaker *erecta* mutant [31,32]. *agb1-1*, which is defective in the β-subunit of the heteromeric G-protein, was included as a mutant of a signalling component acting downstream of *Erecta*[31]. The *V. longisporum* isolate ‘43’ (V43) [33] was used for inoculation experiments.

### Generation of the (Bur×L*er*) RIL population and near-isogenic lines (NILs)

A total of 189 RILs were created originating from an F1 between the ecotypes Bur-0 (♀) and L*er*-0 (♂). A total of 189 F2 plants were propagated via single-seed descent to the F6 generation. All plants were grown in pots of 5 cm diameter in a greenhouse under long-day conditions (16 h light/8 h dark) at 20°C. A total of 94 F6 plants were genotyped and phenotyped in F7. Information about the RIL population will be submitted to the The Arabidopsis Information Resource (TAIR) database [34], and F8 seeds from bulked F7 offspring of the genotyped F6 plants will be made available through the *A. thaliana* stock centres Arabidopsis Biological Resource Center (ABRC) and NASC.

NILs were created by selfing RIL21, which was heterozygous for markers EH2-4 to nga361 on chromosome 2 and homozygous for all other marker loci investigated. NIL5 was selected from RIL21 offspring as homozygous for Bur alleles in the variable region, and NIL9 for L*er* alleles.

### Marker development and analysis

The RILs were genotyped with 73 markers that were polymorphic between the parental ecotypes Bur and L*er*. Among them were 39 simple sequence repeat (SSR) markers, 21 sequence-characterized (SCAR) markers exploiting length polymorphisms between Bur and L*er*, and 12 cleaved amplified polymorphic sequence (CAPS) markers developed on the basis of single nucleotide polymorphisms (SNPs; see Additional file 1). The *erecta* mutation was used as a morphological marker, however, a CAPS marker (BLC2-1) has also been developed to differentiate between the Bur and the L*er* allele of *Erecta*. Fifty-one markers have been published before [8,34,35], and 22 new markers were designed to increase marker density especially in regions of major QTL (Additional file 1). Length polymorphisms between Bur and L*er* were identified using the multiple SNP query tool (MSQT) database [36]. SNPs available through the seqviewer tool of TAIR [37,38] were used for CAPS marker design. Webcutter 2.0 [39] was used to identify differential restriction sites at the sites of SNPs. Primers were designed on the basis of sequence information provided by TAIR.

### Inoculation experiments

Inoculation experiments were performed in the greenhouse or in a growth chamber (Grobank, Mobylux, Germany) under long-day conditions (16 h light/8 h dark) on soil as described previously [8]. Thirty plants were grown in trays measuring 20 × 30 cm. Plants of the same treatment and genotype were arranged in batches of 15 (1/2 tray) or 30 plants (1 tray). Batch replicates were randomized. Plants flowered during the experiment and were grown until the first siliques turned yellow. Two experiments for phenotyping the RIL population were carried out with the same 94 lines (Table 1, experiments 1 and 2). Comparisons between wild type (WT) and *erecta* mutants were performed in two greenhouse experiments (Table 1, experiments 3 and 5), and phytohormone contents were analysed in three experiments (Table 1, experiments 4, 5 and 6). Regarding the analysis of phytohormone contents during development in Bur and L*er* (Table 1, experiment 4), plants were harvested at three different developmental stages: Stage I (early-flowering stage) was analysed in L*er* at 20 days post inoculation (dpi) and in Bur at 31 dpi. Stage II (mid- to late-flowering stage) was analysed at 27 dpi in L*er* and at 38 dpi in Bur. Stage III (onset of silique maturity) was analysed at 31 dpi in L*er* and at 48 dpi in Bur.

**Table 1:** Overview of inoculation experiments (exp.)

**Exp. no.**	**Genotypes**	**Traits**	**Plant replicates control/V43**	**Batch replicates controlcV43**	**Date**	**Environment**
1	Bur, L*er*, 94 RILs	Systemic colonization^1)^, stunting^2)^, chlorosis^3)^, development time^4)^	15/30 for RILs, 90/90 for Bur and L*er*	1/1 for RILs, 6/6 for Bur and L*er*	01/2009-03/2009	Greenhouse
2	Bur, L*er*, 94 RILs	Systemic colonization, stunting, chlorosis development time	15/30 for RILs, 90/90 for Bur and L*er*	1/1for RILs, 6/6 for Bur and L*er*	04/2009-06/2009	Greenhouse
3	La-0, L*er*, Col-0, *er-105*, *er-108*, *er-111*, *er-116*, *er-118*, *agb1-1*	Systemic colonization, stunting, chlorosis	30/300	1/10	01/2010-03/2010	Greenhouse
4	Bur, L*er*	Phytohormone contents in different developmental stages	90-120/90-120	6-8/6-8	12/2009-02/2010	Greenhouse
5	La-0, L*er*, Col-0, *er-105*, Bur	Phytohormone contents	180/180	6/6-7	01/2011-03/2011	Greenhouse
6	Bur, L*er*, NIL5, NIL9	Phytohormone contents	90/90	6/6-7	09/2012-11/2012	Greenhouse
7	Bur, L*er*	Chlorosis	60/60	2/2	08/2008-10/2008	Growth chamber

Assessed parameters per trait for phenotyping: ^1)^a) Percentage of colonized shoot segments, b) fungal DNA content [pg/mg fresh weight], ^2)^a) performance height, b) performance fresh weight, ^3)^a) number of yellow leaves, b) percentage of yellow leaves, c) difference in number of yellow leaves between inoculated and mock-inoculated plants, ^4)^days to flowering in mock-inoculated plants. Plant replicates = number of individual plants; batch replicates = number of batches of 15 or 30 plants growing in a tray.

### Phenotypic analysis

Traits were recorded by different parameters (Table 1). Stunting resistance was measured as “performance height” (mean height_inoculated_/mean height_control_ × 100) and “performance fresh weight” (mean FW_inoculated_/mean FW_control_ × 100). The performance parameters compensate for differences in plant height, and fresh weight between *erecta* mutants and WT. The height was measured between the hypocotyl and the apex of the longest shoot at the beginning of silique maturation when shoots were out-grown. Systemic colonization was determined in apical segments of the main shoot at the onset of silique maturity. To determine the percentage of colonized plants per replicate, one segment per inoculated plant was placed on a malt agar plate as described previously [8], and the percentage of colonized segments was calculated from batches of 15-30 plants. In order to measure the fungal DNA via qPCR, 100 mg of shoot material was cut from segments used for plating and shock-frozen in liquid nitrogen. qPCR using *Verticillium*-specific primers was performed as described [40]. Chlorosis was determined by counting yellow and green rosette leaves. Three different parameters were used to quantify the trait: The number of yellow rosette leaves in inoculated plants was determined as the most direct measure of chlorosis. Furthermore, the percentage of yellow leaves relative to the total rosette leaf number was calculated to express the degree of chlorosis affecting the leaf rosette. As a third parameter, the difference in yellow leaves between inoculated and mock-inoculated plants of the same line is given, thus taking natural senescence into account. Chlorosis in both RIL experiments (experiments 1 and 2, Table 1) was assessed at 17 dpi when the control plants still showed little or no senescence-associated chlorosis, while the *V. longisporum*-inoculated plants were showing chlorotic leaves. In order to determine the time-course of chlorosis, mock-inoculated and inoculated L*er* and Bur plants were assessed every 3-4 days after inoculation in a growth chamber experiment (experiment 7, Table 1). Cotyledons were not included.

### Phytohormone quantification by HPLC-ESI-MS/MS

Approximately 6 cm from the upper half of the stalk were sampled from 15-30 plants of the same treatment and pooled in a single sample. Material was shock-frozen in liquid nitrogen directly after harvest, subsequently lyophilized and ground to a fine powder in a ball mill. An aliquot of 50-100 mg (dry weight) was placed in 2.0 ml tubes and used for phytohormone extraction according to a modified protocol [23]. One ml extraction solvent (20% acetone, 79% H_2_0 and 1% CH_3_COOH, and 2 ng of the deuteriated internal standard D6-salicylic acid, Sigma-Aldrich Co.) was added to each sample. Extraction was performed for 45 min at 4°C on a rotary shaker (160 rpm). Subsequently, each sample was spiked with 1.0 ml diethyl ether (DEE), shortly vortexed, and shaken (160 rpm, 4°C) for an additional 30 min. The sample was centrifuged at 8500 g for 5 min. The upper DEE phase was transferred to a new 1.5 ml tube, and the remaining aqueous phase was re-extracted with 1.0 ml DEE exactly as in the previous step. Both DEE fractions were pooled and dried under vacuum at 30°C, re-dissolved in 200 μl HPLC (high-performance liquid chromatography) solvent (1:1 H_2_O:MeOH containing 7 mM CH_3_COOH), centrifuged once more at 8500 g for 5 min and transferred (180 μl) to an HPLC conical vial (200 μl internal volume, WICOM Germany).

Extracted samples (10 μl) were injected into the HPLC and eluted at 40°C at a flow rate of 0.2 ml/min^-1^. Chromatography was performed on a Kinetex® C18 (100 mm × 2.10 mm with 2.6 μm particle size) column equipped with a C18 guard column purchased from Phenomex Inc. (Aschaffenburg, Germany). The following programme was used for elution: 80% solvent A (water:acetonitrile 95:5) containing 7 mM CH_3_COOH and 20% solvent B (methanol containing 7 mM acetic acid) for 40 s; ramp to 98% B in 50 s; hold for 2 min 20 s followed by re-equilibration to 20% B.

Phytohormones were detected in multiple reaction monitoring mode (MRM) in a triple quadrupole mass spectrometer (LC12000) equipped with an electrospray interface using settings described in [41]. The following mass transitions (collision energy: CE) were used: SA 136.8/93.0 (CE 14.5 eV); d6-SA 140.9/97.0 (18.5 eV), JA 208.9/59.0 (9.5 eV), and ABA 262.8/153.0 (8.0 eV). A calibration curve of the ratio of peak areas of the unlabelled standard to the peak area of the deuterium-labelled standard was used for the quantification of SA. Other phytohormones were quantified with an external calibration curve obtained with pure standard.

### Map construction and QTL analysis

Linkage groups were determined and allele frequencies were tested for segregation distortion with JoinMap [42]. Map construction and QTL analysis was performed with MapManager QTX 20b [43] using the Haldane mapping function. Simple interval mapping was performed scanning the genome in 1 cM-steps. MapManager QTX gives the LRS (likelihood ratio statistic) value to assess the probability of a false positive, where LRS = 4.6 × LOD (likelihood of odds) [44]. LRS significance threshold values for the 37% (suggestive), 95% (significant) and 99.9% (highly significant) genome-wide confidence levels were determined by permutation tests with 10,000 permutations. Confidence intervals for QTL were determined by bootstrap tests which calculate the QTL position for multiple resampled datasets of the original dataset. Epistatic interactions were searched using the “interaction” function of MapManager QTX 20b, testing pairs of markers for a possible interaction component. The confidence criterion for the total effect of a marker pair was set to p = 10^-5^. MapQTL® 6 [45] was also used for mapping to include covariates and cofactors in the QTL analyses. Cofactors were initially specified according to peak positions in interval mapping and selected by backwards elimination using the “Automatic cofactor selection” tool of MapQTL® 6. Maps were visualized using MapChart [46].

### Statistics

All inoculation experiments were performed with batches of at least 15 plants per genotype and treatment. Most inoculation experiments were performed on 6 to 10 batch replicates. Some parameters, such as phytohormone contents, the performance parameters or the percentage of colonized shoot segments, were calculated on a batch basis, whereas some of the chlorosis parameters were calculated on a single-plant basis. Only one batch of 15-30 plants was inoculated per genotype to allow testing of 96 genotypes at the same time in the RIL inoculation experiments. The whole experiment was repeated to confirm the results. See Table 1 for an overview of single-plant and batch replicates. All statistical analyses, as indicated in the Results section, were performed with SPSS 20 [47].

## Results

### QTL controlling resistance traits against *V. longisporum* in the (Bur×L*er*) RIL population

**Figure 1:**
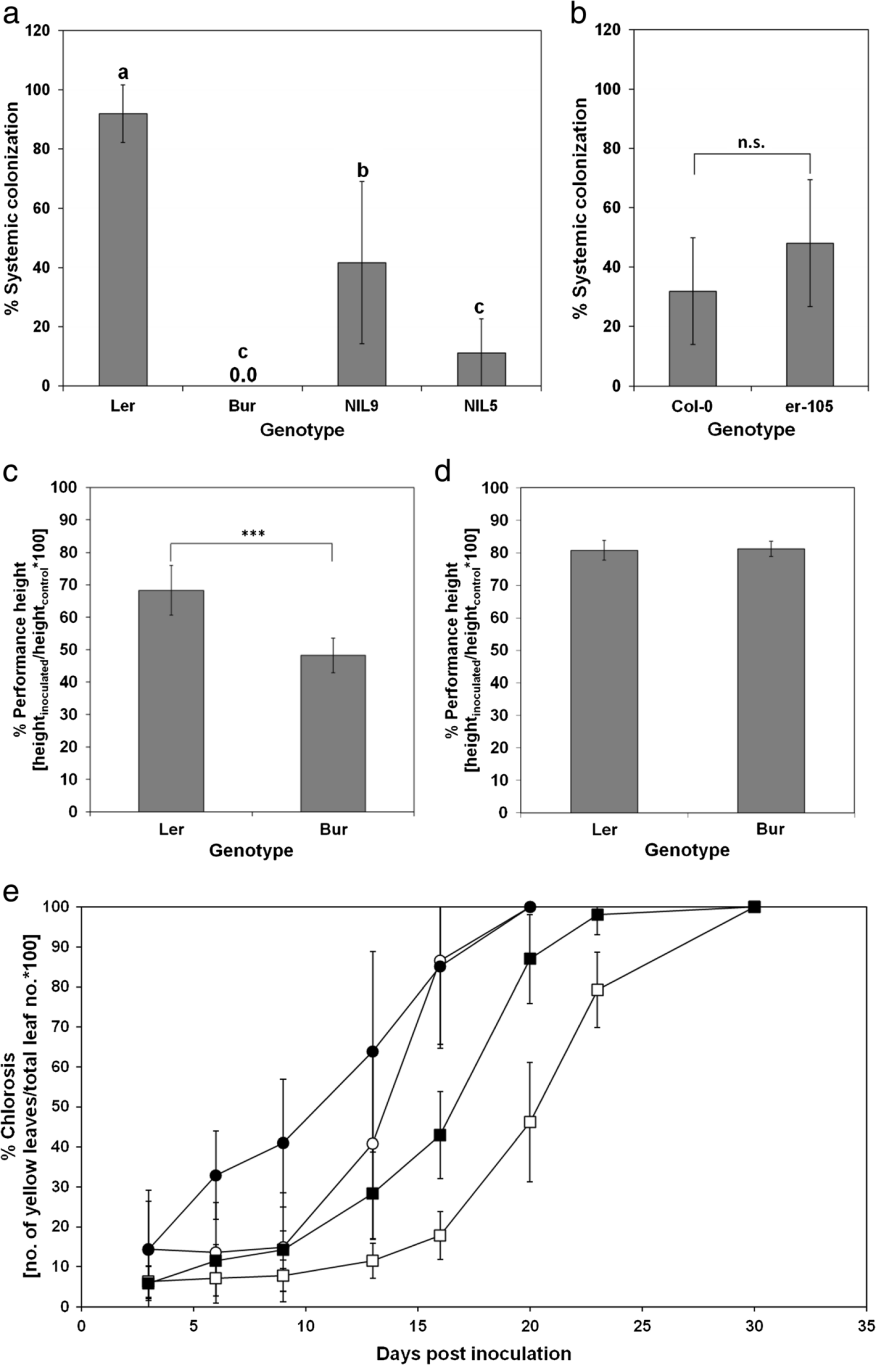
**Phenotypic characterization of different *A. thaliana *genotypes. a,b)** Systemic colonization of **a)** L*er*, Bur and two NILs differing in the *vec1* region (experiment 6; one-way ANOVA and post-hoc Tukey test, p < 0.05, n = 6-7), and **b)** two genotypes differing only in *Erecta* function (experiment 5, *t*-test, p < 0.05, n = 6). **c,d)** Resistance to *V. longisporum*-induced stunting expressed as performance height in **c)** an experiment performed in winter (experiment 1, *t*-test, p < 0.05, n = 6) and **d)** an experiment performed in spring (experiment 2, *t*-test, p < 0.05, n = 6). **e)** Proportion of chlorotic rosette leaves in Bur (squares) and L*er* (circles) after *V. longisporum*-inoculation (closed symbols) and mock-inoculation (open symbols) during the course of a growth chamber experiment. Vertical bars denote standard deviations. Ecotypes and treatments differed significantly from 6 dpi on (p < 0.05; *t*-test; n = 41 for L*er* mock, n = 38 for L*er V. longisporum*, n = 60 for Bur mock, n = 58 for Bur *V. longisporum*).

The parental ecotypes Bur and L*er* differed in important resistance traits against *V. longisporum* (Figure 1). L*er* was susceptible to systemic colonization by the pathogen, whereas Bur showed a high degree of resistance (Figure 1a). Bur, however, was more susceptible to *V. longisporum*-induced stunting than L*er*. This phenotype was suppressed under high-light conditions in the greenhouse during summer (Figure 1c, d). *V. longisporum* promoted chlorosis in both L*er* and Bur compared to mock-inoculated controls. Both natural and *V. longisporum*-induced chlorosis occurred much earlier in the early-flowering ecotype L*er* than in the late-flowering ecotype Bur (Figure 1e).

QTL controlling *V. longisporum* resistance traits were mapped in a new (Bur×L*er*) RIL population. A genetic map was calculated and a physical map was produced using known positions of each marker on the Arabidopsis Genome Initiative (AGI) reference map [48] (Additional file 2). In the genetic map, chromosomes were supported as linkage groups with LOD scores ranging from 4 (chromosome 1) to 10 (chromosome 4). Genetic marker order on each chromosome was the same as for the physical map. The complete map size was 407.3 cM, with an average marker spacing of 5.7 cM and the largest distance between two markers being 17.6 cM. The proportion of heterozygous markers in F6 was 3.65%, which agreed well with a predicted value of 3.125%.

**Figure 2:**
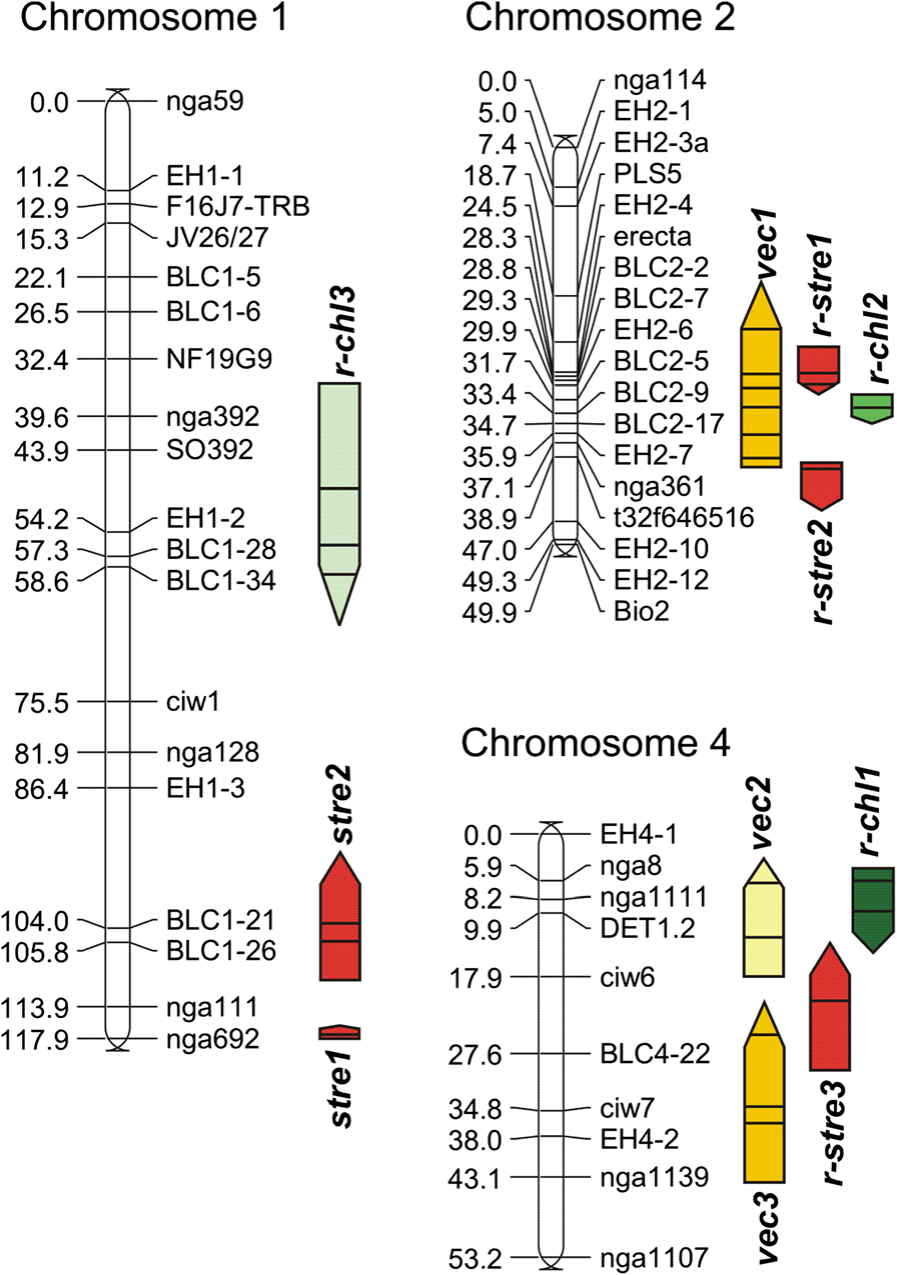
**QTL for *V. longisporum*-related traits in the *A. thaliana *(Bur×L*er*) RIL population.** White bars represent chromosomes with marker positions indicated in cM. Coloured bars delimit the confidence intervals of QTL as follows: Light yellow: Systemic colonization, % colonized shoot segments, exp. 1. Dark yellow: Systemic colonization, % colonized shoot segments, exp. 2. Red: Stunting resistance, performance height, exp. 1. Dark green: Chlorosis, number of yellow leaves, exp. 1. Medium green: Chlorosis, % yellow leaves, exp. 1. Light green: Chlorosis, difference in yellow leaves between mock-inoculated and *V. longisporum*-inoculated plants, exp. 1. Black lines within the bars stand for LRS peak positions. Upward arrows indicate that the paternal ecotype (L*er*) was the origin of the increasing allele; downward arrows stand for Bur as the source of the increasing allele. QTL from the most representative dataset are displayed in Figure 1. QTL names are written next to the bars. For reproducibility and further QTL information, see Additional file 3.

QTL were detected for all three resistance traits (Figure 2, Additional file 3). QTL controlling *Verticillium****c*olonization** (*vec*) were detected on chromosomes 2 and 4, QTL for ***st*unting***re*sistance (*stre* and *r-stre*) on chromosomes 1, 2 and 4, and QTL for resistance against *V. longisporum*-induced ***chl*orosis***(r-chl*) also on chromosomes 1, 2 and 4. QTL were named according to the nomenclature used previously [8]. QTL exclusively discovered in the RIL population were prefixed with an “r-”, indicating their origin from RIL mapping. All parameters characterizing the traits showed either a normal or a multi-modal frequency distribution, both of which can occur in traits controlled by several QTL (Additional file 4).

All *vec* QTL controlling systemic **colonization** consisted of arrays containing several LRS peaks depending on the parameter and the inoculation experiment (Figure 2, Additional file 3). The map positions of the peaks were reproducible for both parameters (plating assay and qPCR) and in both inoculation experiments (Additional file 3). *vec1* explained up to 19.3% of the trait variance, depending on the parameter and the experiment, *vec2* up to 15% and *vec3* up to 17.3% (Additional file 3). The *vec1* QTL could be confirmed in NILs that differed in a ~ 3 megabase-segment on chromosome 2 comprising *vec1*. NIL9 with L*er* alleles in the variable region was more susceptible to systemic colonization than NIL5 with Bur alleles in the respective region (Figure 1a). All *vec* alleles increasing the degree of colonization were of L*er* origin. Thus, colonization resistance was entirely conferred by the Bur alleles of the respective QTL. The co-localisation of colonization and developmental QTL on chromosome 4 (Additional file 3) suggested an impact of development on systemic colonization, although all assessments were made at defined developmental stages. Development parameters were used as covariates in interval mapping or multiple QTL mapping (MQM) with MapQTL. Using the developmental parameters as a covariate, all *vec* QTL on chromosome 4 vanished, and *vec1* on chromosome 2 remained as the only chromosomal region showing a significant effect on this resistance trait. Co-factor selection in the QTL region on chromosome 2 and application of the selected co-factors in MQM confined the QTL region controlling the degree of systemic colonization (both parameters) to a fragment between markers EH2-6, BLC2-2 and *erecta*, spanning approximately 1.6 cM.

QTL for **stunting** resistance could only be detected with the parameter “performance height” and not with “performance fresh weight”. Both QTL on chromosome 2 were detected in both inoculation experiments; the QTL on chromosomes 1 and 4 were only detected in experiment 1, which was performed in the winter. *stre2* co-localised with marker BLC1-26, which is located in the *Wakl9* gene, and explained up to 22.9% of the trait variance. *stre1*, explaining 16.8% variance, was also located in the vicinity of a *Wakl* gene (*Wakl22*/*rfo1*). *r-stre1* near marker *erecta* on chromosome 2 explained approximately 30% of the trait variance in experiment 1 (Figure 2, Additional file 3). The stunting resistance alleles of the QTL on chromosome 2 were of Bur origin, whereas the stunting resistance QTL on chromosomes 1 and 4 were contributed by L*er*. The fact that both parents contributed stunting resistance QTL explains the strong transgressive segregation of the trait (Additional file 4). As for the degree of systemic colonization, the development apparently had an impact on the degree of stunting. Including development traits as a covariate in mapping reduced the number of QTL controlling this trait by eliminating the QTL *r-stre3* on chromosome 4, which had been detected in experiment 1.

Significant QTL for **chlorosis** parameters (*r-chl*) were only detected in experiment 1 (Figure 2, Additional file 3). *r-chl1* on chromosome 4 was the strongest QTL explaining up to 29.3% of the trait variance. *r-chl2* on chromosome 2 explained 11.7% and *r-chl3* on chromosome 1 up to 14.8%. Surprisingly, all alleles conferring resistance to chlorosis were of L*er* origin. Two QTL accelerating flowering time of L*er* origin were found in the same region (*dt1* and *dt2*, see Additional file 3). In general, a later onset of flowering is expected to correlate with a later onset of senescence. Furthermore, using development traits as a covariate did not affect *r-chl1* on chromosome 4. Both facts are strong evidence that QTL have been mapped that were specific for *V. longisporum*-induced chlorosis and not QTL controlling development.

Epistatic interactions between marker loci could not be reproduced between the tests. Regarding the colonization data determined by the plating assay in experiment 1, a significant interaction between marker loci BLC2-7 on chromosome 2 and nga1111 on chromosome 4 was detected. Both loci lie within the confidence intervals of *vec1* and *vec2*, respectively. In summary, it could be shown that QTL from both parents controlled different resistance traits against *V. longisporum* in the (Bur×L*er*) RIL population.

### *V. longisporum*-induced stunting was controlled by ERECTA signalling

**Figure 3:**
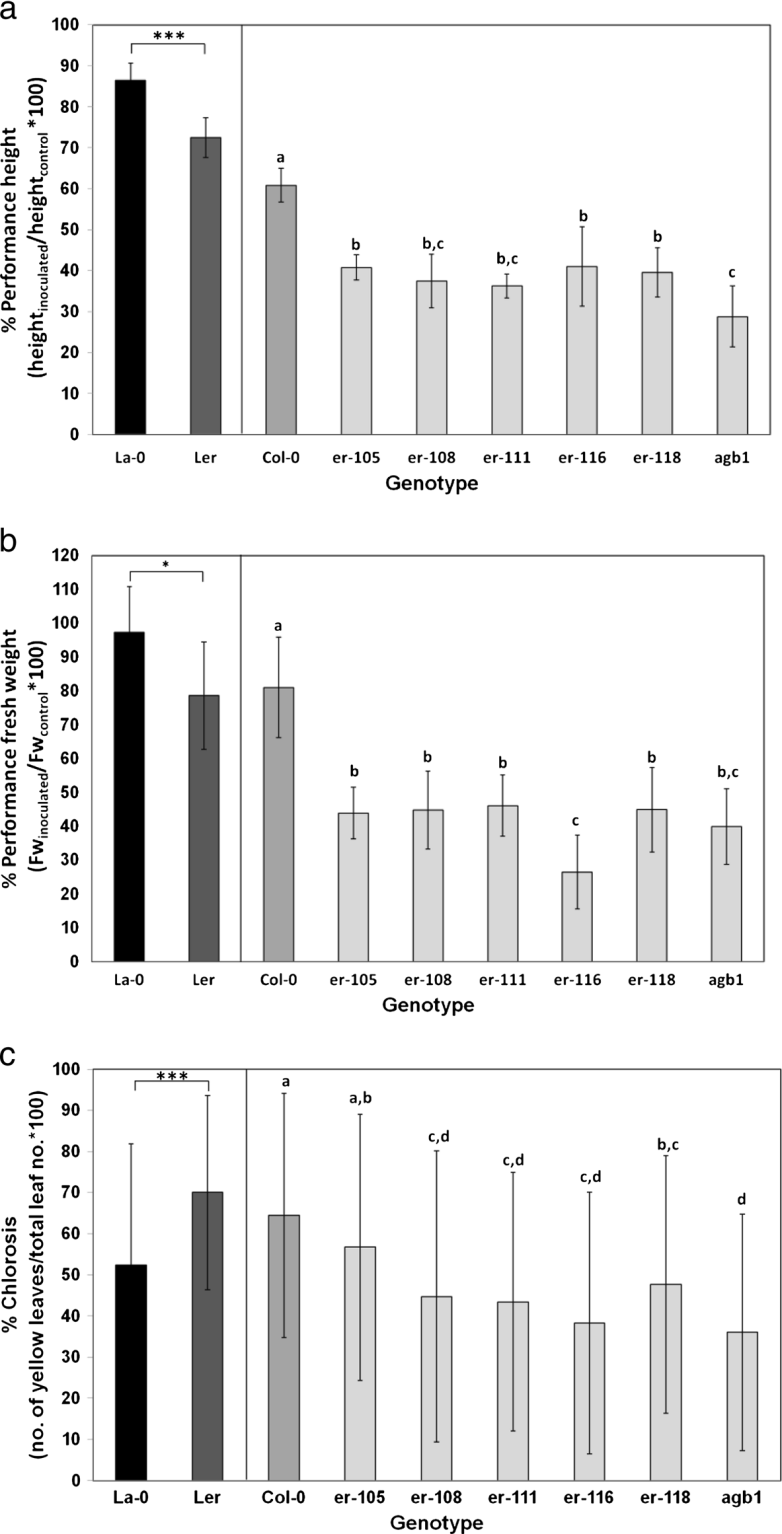
**Reaction of *Erecta *signalling mutants and corresponding WT lines to *V. longisporum*.** La-0 is the corresponding WT for L*er*, Col-0 for *er-105*, *er-108*, *er-116*, *er-118*, and *agb1-1*. Different WT/mutant combinations are separated by a vertical bar. Recorded parameters are **a)** performance height, **b)** performance fresh weight and **c)** % chlorotic leaves in inoculated plants. N = 10 for **a,b)**, N between 91 and 260 for **c)**. Significance of La-0/L*er* differences was tested by t-tests; differences between lines with Col-0 background by one-way ANOVA and subsequent multiple comparisons (Tukey test). Means marked with different letters differed significantly at p < 0.05. Vertical bars denote standard deviations.

The strong stunting resistance QTL *r-stre1* co-localised with the morphological marker *erecta. Erecta* encodes for a receptor-like kinase with many functions in plant development and also in QDR [49]. The function of this protein in L*er* is disrupted [32], while Bur contains a functional *Erecta* allele. Inoculation experiments were performed on *erecta* mutants and their respective WT ecotypes having a functional *Erecta* allele to investigate whether a functional *Erecta* pathway increases resistance. L*er* was compared with the La-0 ecotype. Col-0 was compared with *erecta* mutants in the Col-0 background. The *agb1-1* mutant in the Col-0 background that is defective in the β-subunit of heteromeric G-protein was included representing another component of the *Erecta* signalling pathway [31]. All *erecta* mutants and the *agb1-1* mutant were significantly more stunted under *V. longisporum* challenge than their respective WT lines: The parameters “performance height” and “performance fresh weight” were decreased in all mutants compared to WT (Figure 3a, b). The weaker *er-116* mutant did not perform better than the strong mutants. The developmental phenotype of *er-116*, however, was clearly attenuated: The mean height of the controls was 22.3 cm compared to 16.5 cm in the strong *erecta* mutants. A functional *Erecta* did not contribute significantly to colonization resistance in any of the two backgrounds (Figure 1b, Additional file 5), despite the strong colonization QTL *vec1* that was localized near *Erecta*. Because one QTL for chlorosis (*r-chl2*) was localized in the *Erecta* region, it was investigated whether loss of *Erecta* function influences chlorosis. The level of chlorosis in Col-0 was higher than in the *erecta* mutants with Col-0 background (Figure 3c), suggesting a chlorosis-promoting effect of *Erecta*. However, the effect of *Erecta* was opposite in La-0/L*er*: La-0 suffered less chlorosis than L*er* (Figure 3c). Thus, a background-specific effect of *Erecta* on chlorosis must be assumed.

It was demonstrated that *Erecta* underlay stunting resistance QTL *r-stre1* and that a functional ERECTA signalling pathway mediated stunting resistance in *A. thaliana*.

### SA and low light were associated with *V. longisporum*-induced stunting

**Figure 4:**
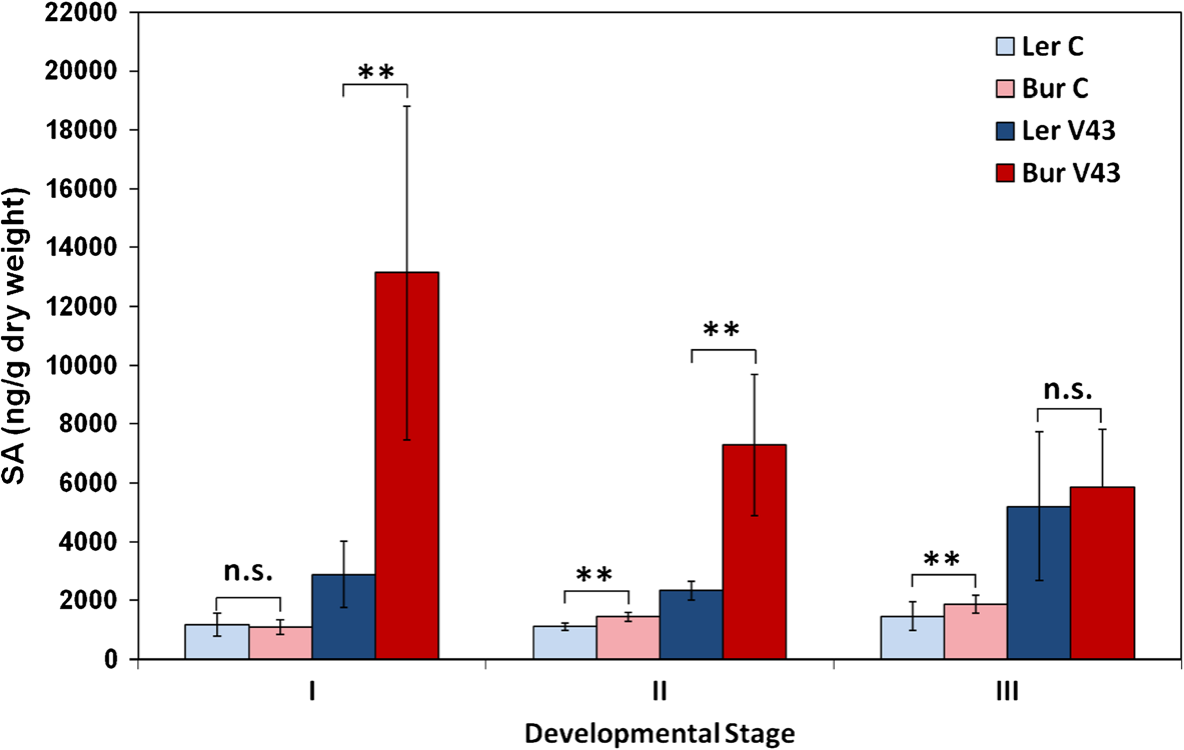
**Contents of the phytohormone SA in L*er *and Bur, mock-inoculated and inoculated, at different developmental stages.** I: early-flowering stage, II: mid-flowering stage, III: beginning of silique maturity. N = 8. Significance levels refer to differences of means between the ecotypes **within one treatment** (mock- and *V. longisporum*-inoculated, respectively) as determined by t-tests. Differences between mock- and *V. longisporum*-inoculated plants were always significant. V43 = *V. longisporum* isolate 43.

SA content increased in *A. thaliana* stalks after infection with *V. longisporum* in both Bur and L*er* (Figure 4). SA levels were strongly increased in Bur at early- to mid-flowering stages, while SA showed a mild but significant increase in L*er* with the advance of flowering (Figure 4). SA hyperinduction was neither associated with *vec1* nor with *Erecta*, as could be shown in NILs and *erecta* mutants (Additional files 6 and 7). Furthermore, Bur showed high susceptibility to *V. longisporum*-induced stunting in winter experiments (Figure 1c). This season-dependent susceptibility could be attributed to the Bur alleles of QTL *stre1*, *stre2* and *r-stre3*, the first two co-localising with *Wakl* genes. Greenhouse experiments were always performed in long-day conditions with additional illumination at temperatures between 20 and 25°C. However, the intensity, dosage and spectral composition of ambient light were major factors differing between spring/summer and winter experiments: Quantum flux densities of artificial illumination alone ranged between 80 and 100 μmol*s^-1^*m^-2^, whereas additional natural daylight provided between 200 and 400 μmol*s^-1^*m^-2^.

It is hypothesized that SA induction and low light caused *V. longisporum*-induced stunting mediated by the seasonally influenced QTL *stre1*, *stre2* and *r-stre3* (see Discussion).

### ABA and JA response to *V. longisporum* infection was controlled by *vec1*

**Figure 5:**
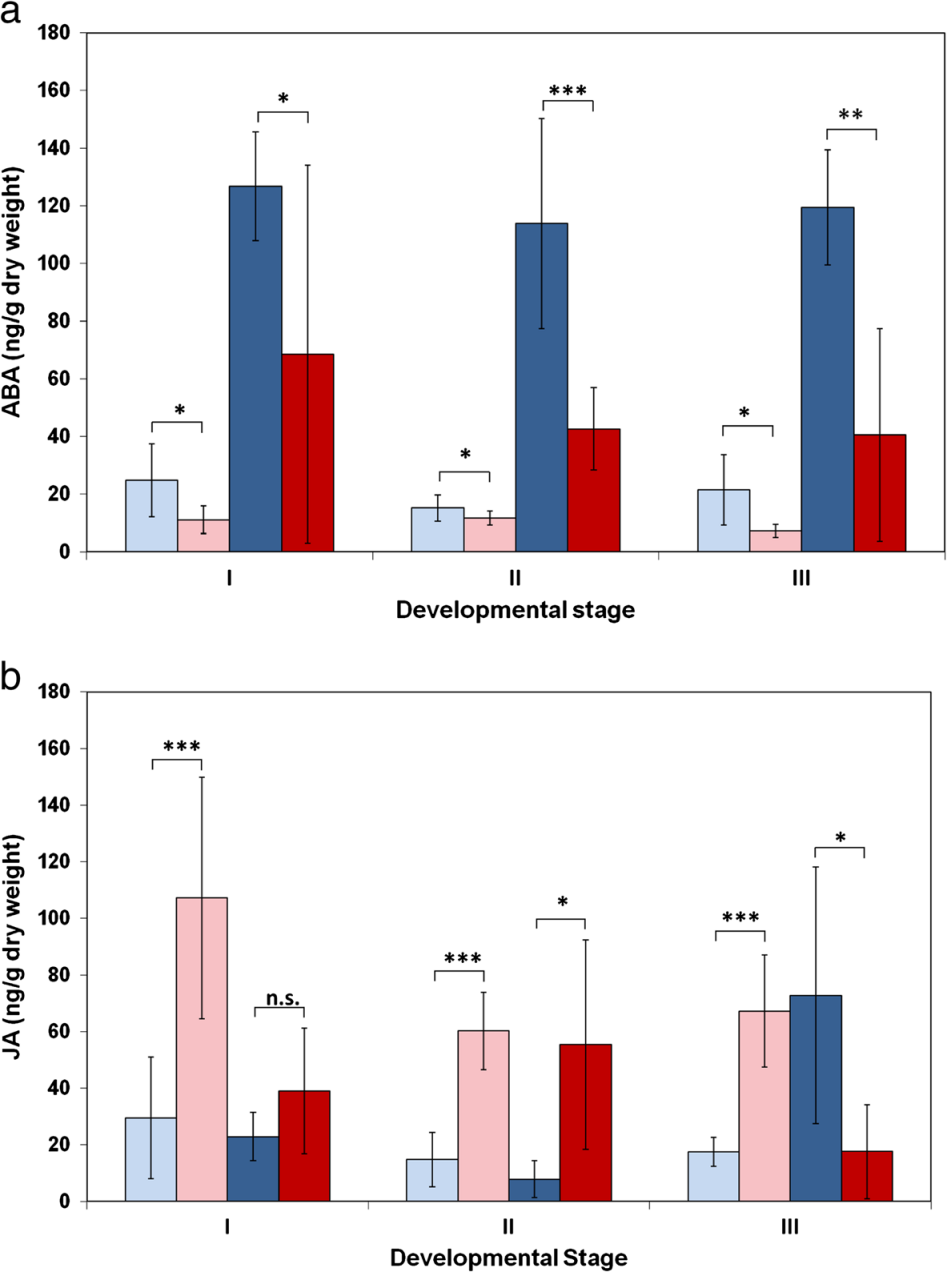
**Contents of the phytohormones a) ABA and b) JA in L*er *and Bur, mock-inoculated and inoculated, at different developmental stages.** Light blue: L*er* control, dark blue: L*er* inoculated, pink: Bur control, red: Bur inoculated. I: early-flowering stage, II: mid-flowering stage, III: beginning of silique maturity. N = 8. Significance levels refer to differences of means between the ecotypes within one treatment (mock- and *V. longisporum*-inoculated, respectively) as determined by t-tests. Differences between mock- and *V. longisporum*-inoculated plants were significant except for ABA in Bur at stage III, JA in L*er* at stages I and II and JA in Bur at stage II (p < 0.05, *t*-test).

**Figure 6:**
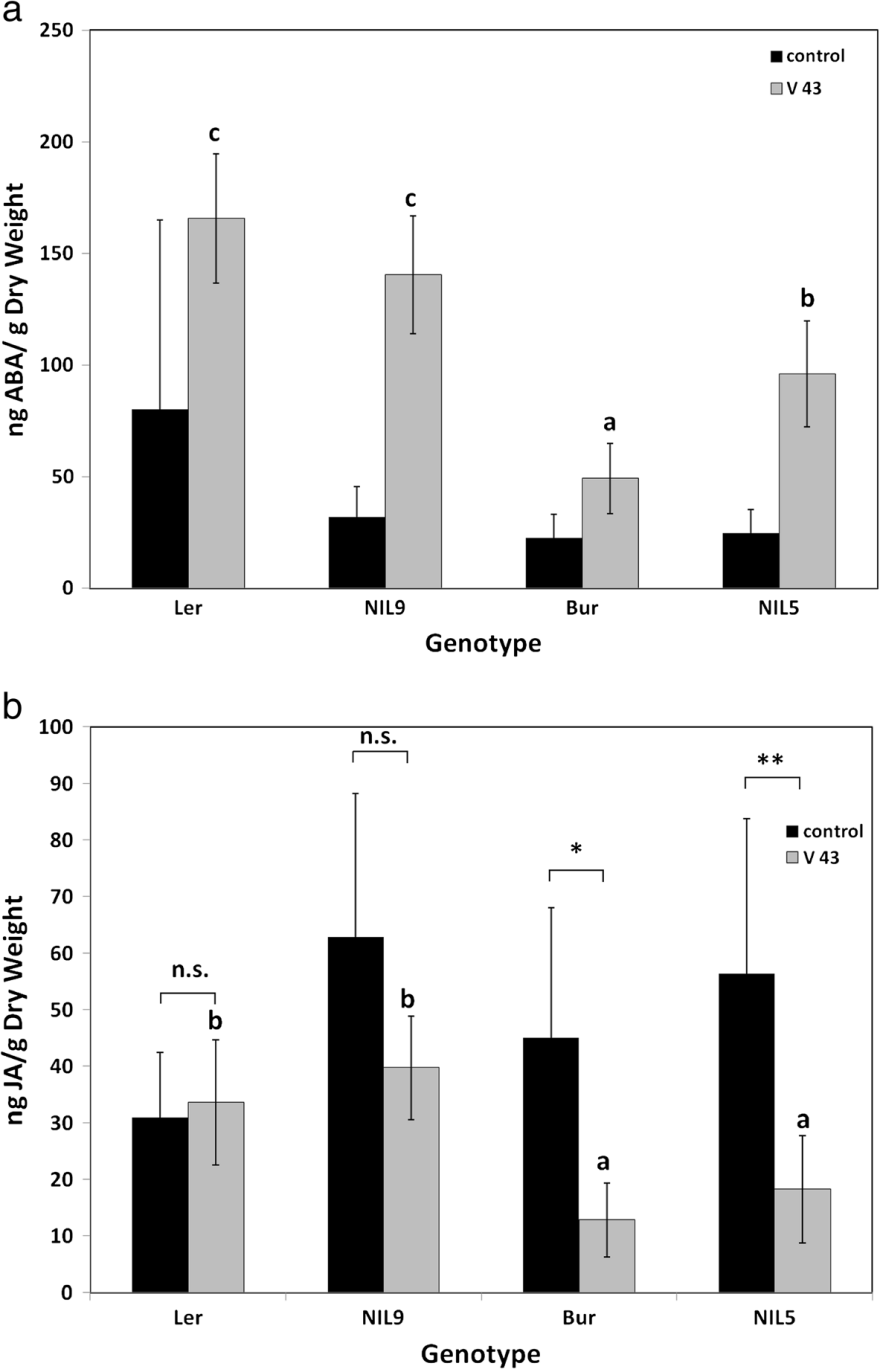
**Contents of the phytohormones a) ABA and b) JA in Bur*, *L*er *and two near-isogenic lines.** NIL9 contained L*er* alleles in the variable region and NIL5 contained Bur alleles. **a)**: Differences between mock- and *V. longisporum*-inoculated plants were significant (n = 6-7, p < 0.05). **b)** Asterisks refer to the significance level of differences between mock-inoculated and *V. longisporum*-treated plants within one genotype (*t*-test, n = 6). Mean contents of inoculated plants marked with different letters differed significantly at p < 0.05. Mean phytohormone contents of controls did not differ significantly (one-way ANOVA and post-hoc Tukey test). Vertical bars denote standard deviations. V43 = *V. longisporum* isolate 43.

Bur and L*er* differed in their ABA and JA responses to *V. longisporum*: ABA contents were much higher in L*er* than in Bur upon infection (Figures 5 and 6). Changes of JA levels were opposite in L*er* and Bur at the maturity stage: the JA level in L*er* stayed the same or increased, whilst the JA levels in Bur dropped in infected plants compared to mock-inoculated plants (Figures 5 and 6). Analysis of ABA and JA content in NILs at the maturity stage showed that this difference was associated with the genomic region containing *vec1* and *Erecta* on chromosome 2 (Figure 6). NIL9, which was homozygous for L*er* alleles in the variable region, showed strong ABA induction and no JA decrease – a reaction that was similar to the one in L*er*. NIL5 with Bur alleles in the variable region showed a reaction of both phytohormones that resembled Bur: ABA increased less strongly and JA decreased in infected plants (Figure 6). The characteristic phytohormone response patterns corresponded to high systemic colonization in L*er* and NIL9 and low colonization in Bur and NIL5 (Figure 1a). No effect of *Erecta* on ABA and JA levels in *V. longisporum*-infected plants was found when *erecta*/WT comparisons were made for ABA and JA contents (Additional file 7). ABA and JA signalling pathways are candidates for the control of systemic colonization by *V. longisporum* in *A. thaliana*.

## Discussion

Considerable progress has been made in understanding the molecular interactions of *V. longisporum* with its cruciferous hosts at a molecular level during recent years. Many genes and pathways have been shown to be involved, revealing an enormous complexity. The present study has been designed to further disentangle the complex network and to identify new components of genetic variation contributing to quantitative resistance to *V. longisporum*.

Our results corroborate the view that different resistance traits are controlled by different pathways and can be inherited independently. A plant showing a high degree of colonization resistance may still be susceptible to stunting. No single master mechanism controlled QDR in *A. thaliana*. Nevertheless, a higher resistance level can be achieved by combining a few well-defined QTL. Some components of the physiological basis of resistance were elucidated.

Phenotypic variation for colonization resistance and stunting resistance was assigned to specific QTL that have been partly detected before, but were mapped with much higher resolution in the RIL population. *vec1*, *vec2* and *vec3* were found to control systemic colonization by *V. longisporum* in an F2/F3 population originating from the same parents [8] and could be reproduced in the present study. RIL mapping revealed a high complexity of the *vec* QTL. Multiple, reproducible LRS peak positions suggest complex loci in which more than one gene contributed to the effect. The genes underlying the *vec* QTL are still unknown, but recent results strongly suggest that genes involved in the phenylpropanoid pathway underlie *vec3*[20]: The genes *cad5*, *cad8* and *ugt84a3*, encoding for cinnamyl alcohol dehydrogenases and an UDP-glycosyl-transferase, are all located on chromosome 4 close to *vec3*. Soluble phenylpropanoids have been shown to play a role in defence against *V. longisporum*[20]. An impact of development on resistance could be demonstrated. Systemic colonization of the upper parts of the plant starts with the onset of flowering in *A. thaliana* and *Brassica* spp. [8,9]. Direct or indirect signals that induce or promote flowering possibly affect the development of *V. longisporum* as well, for example, by stimulating the formation of mobile conidia or by directing growth of fungal hyphae. The loss of the *vec2* and *vec3* QTL when using development as a covariate in QTL analyses indicates that the developmental differences on a physiological basis were still strong enough to cause the detection of these QTL, even though all plants had been assessed at the same phenotypic developmental stage. Developmental implications also played a role in stunting. The stunting resistance QTL on chromosome 4, *r-stre3*, similar to *vec2* and *vec3*, disappeared when development was used as a covariate in MQM mapping.

The stunting resistance QTL on chromosomes 1 (*stre1* and *stre2*) and 4 (*r-stre3*) depended on the season: They protected against stunting only during winter, which is consistent with prior observations for *stre1* and *stre2*[8]. Despite complementary lighting in the greenhouse, the development of *A. thaliana* was prolonged during winter (experiment 1) and developmental differences were more pronounced. Differences in light intensity and/or quality due to more natural illumination during summer are likely to cause these reactions. The strongest QTL on chromosome 1, *stre2*, co-localised with the *Wakl9* gene, encoding a wall-associated kinase (WAK)-like protein [50]. A candidate for *stre1* at the bottom of chromosome 1 is *Rfo1* (*Wakl22*), encoding another WAK-like protein which has already been shown to play a role in *V. longisporum* resistance [16]. Since *V. longisporum* interacts with the host plant primarily in the apoplast [51], perception of pathogenesis-related molecules by cell-wall associated proteins may be crucial, and WAK proteins have been shown to mediate such perceptions [52]. Interestingly, several *Wak* genes have been shown to be induced by SA [53]. An SA induction of *Wakl* genes has not yet been shown, but it would correspond well with the early and strong SA induction detected in Bur. Increased SA levels have been observed to cause stunted growth also without disease [54]. The stunting effect of SA has been shown to be partially reverted by high-light conditions [55]. The combined effects of high levels of SA and lighting differences could explain the pronounced *Verticillium*-induced stunting mediated by the *stre1* and *stre2* alleles of Bur origin during winter. High levels of SA and its glucoside were also found in the xylem sap of *V. longisporum*-infected *B. napus* and were correlated to the degree of stunting and the amount of pathogen DNA [23]. Stunting is a common symptom in greenhouse or growth chamber experiments with *V. longisporum* occurring already at the rosette stage, but is never seen in infected field crops. Light intensity and quality is a major difference between field and greenhouse. This suggests that *V. longisporum*-induced stunting under experimental conditions also depends to a certain extent on the combination of high SA levels with artificial lighting.

The QTL controlling chlorosis seemed to be less affected by genes controlling development. The opposite has been reported in the literature [10]: A QTL delaying development and mediating resistance against *Verticillium*-induced chlorosis, *Vet1*, has been identified in *A. thaliana* ecotype C24. Interestingly, the *r-chl1* QTL on chromosome 4 co-localised with *Vet1*. The close proximity of several genes controlling the transition to flowering in this region (*fri, cry1/hy, det1, ted1*) complicates an interpretation of these results.

In the present study, *Erecta* has been shown to underlie the stunting resistance QTL *r-stre1* on chromosome 2, which explained a large part of the trait variation observed. Receptor-like kinases (RLKs) are often involved in controlling developmental processes or mediating disease resistance reactions [56]. The only *Verticillium* resistance gene identified so far, *Ve1*, also belongs to the RLK family [57,58] and has been shown to recognize fungal effectors [59]. The leucine-rich repeat receptor-like Ser/Thr kinase ERECTA is an example of a signalling molecule controlling both developmental processes and QDR in *A. thaliana*[49]. *erecta* mutants show altered organ development resulting in compact growth [32,60]. Cell proliferation [61] and stomatal patterning [62] were also shown to be controlled by *Erecta*. Additionally, several resistance traits are reported to be controlled by *Erecta* in different pathosystems: Growth of the bacterial pathogen *Ralstonia solanacearum* was inhibited and wilt symptoms were reduced in *A. thaliana* plants with a functional *Erecta* gene compared to *erecta* mutants [63]. Infection with the necrotrophic fungus *Plectosphaerella cucumerina* resulted in more chlorosis and necrosis in *erecta* mutants compared to the respective WT accessions [64]. Enhanced susceptibility of *erecta* mutants, leading to enlarged leaf lesions, has also been reported for infection with the oomycete *Pythium irregulare*[65]. These results illustrate the close interconnection between ERECTA, development and QDR. Hence, it is not always clear whether altered disease resistance is a consequence of the developmental changes or a direct effect of ERECTA signalling. Studying other signalling components of the ERECTA pathway can shed light on this question. Plants defective in *Agb1* are morphologically distinct from *erecta* mutants [31], but defective in the same signalling pathway. In the present study, *agb1-1* mutants were at least as susceptible to *V. longisporum*-induced stunting as the *erecta* mutants. This supports the view that a functional ERECTA pathway mitigates the stunting effect of *V. longisporum* infection independently of its effect on morphology.

Resistance to systemic colonization was not enhanced by a functional *Erecta* gene in La-0 compared to L*er*. This is strong evidence that *Erecta*, although it is located in *vec1*, is not involved in mediating resistance to systemic colonization by *V. longisporum*. A tendency towards stronger colonization of *erecta* mutants in the moderately susceptible Col-0 background compared to WT was never significant, and is interpreted as an indirect effect of the reduced plant height caused by *erecta. V. longisporum* may reach the apex of a shorter shoot more easily. This view is corroborated by the fact that the long stalks of the *agb1-1* mutant were very poorly colonized, thus behaving very differently from the *erecta* mutants, despite the fact that both AGB1 and ERECTA act in the same signalling pathway.

Altogether, *Erecta* has been proven to be a source of natural genetic variation in quantitative resistance not only to *V. longisporum*. Not much is known so far about the allelic variation of *Erecta* beyond the level of complete loss of function and its consequences for disease resistance in natural accessions. A comprehensive analysis of *Erecta*, its structural and regulatory variability and its homologues in cruciferous crop plants would, therefore, be desirable.

ABA and JA contents differed in a genotype-specific way that correlated with the rates of fungal colonization. It is not known whether *V. longisporum* produces ABA itself, which could explain the high levels in heavily colonized plants. In *B. napus*, however, *V. longisporum* infection did not increase ABA levels in xylem sap [23]. The sampling was not fully comparable in both studies, as different parts of the stalk were sampled and the samples in *B. napus* were taken at an earlier developmental stage. The results in *B. napus* make it more likely that different ABA levels depended on the host genotypes instead of fungal biomass.

The role of ABA signalling in *V. longisporum* resistance is complex. The ABA-deficient *aba2-1* mutant was highly susceptible to *V. longisporum*-induced stunting, but other ABA signalling mutants were unaffected, indicating that a specific function of ABA2 was involved in the response observed that did not require ABA in general [16]. From the many functions of ABA in development and disease, different effects can be assumed in the context of *Verticillium* disease. ABA has been shown to influence plant defence reactions in various ways [66]. In most cases, ABA increased susceptibility to pathogens due to suppression of SA synthesis [67] and/or antagonism with jasmonate-ethylene signalling [68]. However, ABA can also stimulate JA biosynthesis and increase resistance [65]. ABA has important functions during seed development, such as a trigger for the acquisition of storage molecules during cell enlargement [69]. ABA plays a significant role in the induction of senescence [70]. A major effect of *V. longisporum* on *B. napus* in the field is the induction of premature ripening, which can be recognized by chlorotic stems and which is leading to reduced seed size. Increased levels of ABA might support the deviation of mobilised nutrients to foster the growth of fungal biomass. Furthermore, it is possible that clogged vessels after *V. longisporum*-infection induce increased ABA levels as a result of drought stress. However, it has recently been shown that *V. longisporum* infection can even increase drought tolerance of the host as a consequence of *de-novo* xylem formation [29]. Studying xylem trans-differentiation in susceptible and resistant genotypes could clarify the relevance of xylem formation for resistance.

JA contents decreased after infection with *V. longisporum* in Bur and the NIL that contained Bur alleles in the region of *vec1*. Plant-pathogenic *Verticillium* species are regarded as hemibiotrophs with a necrotrophic phase during the late stages of infection. Defence responses against necrotrophic pathogens are often induced by JA [71]. Accordingly, JA-deficient tomato plants have been shown to be more susceptible to *Verticillium dahliae* than WT plants [72]. In the present study, however, the colonization-resistant ecotype Bur was characterized by a decrease in JA levels after infection. Since JA is also involved in *A. thaliana* leaf senescence [73], *V. longisporum* possibly benefits from senescence processes induced by JA and ABA, which would suggest a stimulation of hormone production by the fungus. Alterations of senescence-like processes were also postulated to underlie increased resistance of the *A. thaliana* JA-receptor mutant *coi1* against *V. longisporum* colonization [24]; however, this disease-promoting effect of *Coi1* has been shown to be JA-independent. These results confirm that fine-tuning of a resistance reaction by cross-talk of phytohormone signalling pathways is highly individual for each pathosystem [74]. In addition, possible manipulations of the host’s hormone status by the pathogen for its own benefit should be considered.

## Conclusions

Phytohormone signalling processes have been demonstrated to be subject to allelic variation and underlie QDR against *V. longisporum* in natural *A. thaliana* accessions. ERECTA, SA, ABA, and JA signalling has been shown to mediate an ecotype-specific response of *A. thaliana* to *V. longisporum* infection. The ecotype-specific differences for ABA and JA contents were mediated by the same genomic region on chromosome 2 that also controlled systemic colonization by *V. longisporum*. This region contains the major QTL controlling systemic colonization, *vec1*, and also *Erecta*. As colonization resistance was shown to be independent of *Erecta*, this region should contain other gene(s) that control the degree of fungal colonization. This type of resistance is likely to depend on ABA and JA signalling, as both NILs differed for these hormone contents in the same way as the parental lines that contributed the respective alleles in the polymorphic region. Ongoing studies on differential gene expression depending on *vec1* should allow one to draw a more comprehensive picture of the defence reactions leading to colonization resistance. Furthermore, cloning of a gene that controls this reaction should be feasible by combining map-based confinement of the QTL region with expression analysis. It was demonstrated that different resistance traits were controlled independently on a physiological basis, but were still genetically linked. Understanding the biological basis of phenotypic variation in *A. thaliana* with respect to *V. longisporum* resistance will provide new approaches for implementing durable resistance in cruciferous crops.

## Competing interests

The authors declare that there are no competing interests.

## Authors’ contributions

EH planned all the plant experiments, supervised experiment 3, carried out all other plant experiments, analysed the data, and drafted the manuscript. PK provided the qPCR data on fungal biomass and helped to draft the manuscript. RS provided all data on phytohormone contents and helped to draft the manuscript. AT carried out experiment 3, collected the data and helped with their analysis. ED conceived of the study, participated in its design and coordination, participated in QTL analysis, and helped to draft the manuscript. All authors read and approved the final manuscript.

## Supplementary Material

Additional file 1 **Marker information.** Lists all markers that have been used for genotyping the (Bur×L*er*) RIL population. It consists of two tables. **Table S1** is an overview of all markers used, their type, chromosomal location, and their source. In **Table S2**, the technical information of all new markers is listed. This includes primer information, melting temperature of primers, information on the polymorphism, and the PCR protocol. Click here for file

Additional file 2 **Genetic and physical map.** Shows the genetic map and the physical map of the (Bur×L*er*) RIL population. The genetic map shows marker distances for all chromosomes in cM, the physical map shows the location of markers in kilobases (kb) according to the AGI map [48]. Click here for file

Additional file 3 **QTL information.** Lists information for all QTL detected in the present study with 94 (Bur×L*er*) RILs in two separate experiments. QTL for the traits systemic colonization, stunting resistance and resistance to chlorosis detected with different parameters are listed in a table. The chromosomal location, the LRS peak position(s), the LOD score, the additive component, the explained trait variance, and allelic means are given for each QTL. Click here for file

Additional file 4 **Frequency distribution of phenotypic data.** Shows the frequency distribution histograms for the phenotypic data used in QTL mapping. Histograms are shown for the parameters “% colonized shoot segments”, “pg *Verticillium* DNA/mg fresh weight”, “performance height”, “Mean number of yellow leaves in *V. longisporum*-inoculated plants”, “Mean number of yellow leaves related to total rosette leaf number”, and “Mean difference in yellow leaves between inoculated and mock-inoculated plants”. Click here for file

Additional file 5 **Systemic colonization of *Erecta* signalling mutants and corresponding WT-lines.** Contains a bar chart visualizing systemic colonization of *erecta* mutants, *agb1-1* mutant and the corresponding WT genotypes. It provides evidence that *Erecta* is not involved in mediating resistance to systemic colonization by *V. longisporum*. Click here for file

Additional file 6 **SA contents in Bur, L*er* and NILs.** Contains a bar chart visualizing SA contents in Bur, L*er* and two NILs differing in the *vec1* region. The data provide evidence that SA hyperinduction, which is characteristic for Bur, is not associated with the *vec1* locus. Click here for file

Additional file 7 **Phytohormone contents of *erecta* mutants, corresponding WT-lines and Bur.** Contains three bar charts visualizing SA, ABA and JA contents of *Erecta* signalling mutants and corresponding WT lines after mock-inoculation and *V. longisporum*-infection. Additional file 7 provides evidence that differences in phytohormone response to *V. longisporum* between Bur and L*er* are not caused by *Erecta*. Click here for file
