# Supplementary material for: ERECTA, salicylic acid, abscisic acid, and jasmonic acid modulate quantitative disease resistance of Arabidopsis thaliana to Verticillium longisporum
Source: BMC Plant Biol. 2014 Apr 1;14:85. doi: 10.1186/1471-2229-14-85 (PMC4021371; doi:10.1186/1471-2229-14-85)
Supplement: Additional file 1 — Marker information. Lists all markers that have been used for genotyping the (Bur×Ler) RIL population. It consists of two tables. Table S1 is an overview of all markers used, their type, chromosomal location, and their source. In Table S2, the technical information of all new markers is listed. This includes primer information, melting temperature of primers, information on the polymorphism, and the PCR protocol. [file 1471-2229-14-85-S1.pdf]

Additional File 1: Marker information

Table I: Overview of markers used for genotyping the (Bur×L*er*) RIL Population

Marker Name	Marker Type	Chr.	Source
nga59	SSR	1	TAIR
F16J7-TRB	SSR	1	TAIR
JV26/27	SSR	1	TAIR
NF19G9	SSR	1	TAIR
nga392	SSR	1	TAIR
SO392	SSR	1	TAIR
ciw1	SSR	1	TAIR
nga128	SSR	1	TAIR
nga111	SSR	1	TAIR
nga692	SSR	1	TAIR
nga114	SSR	2	TAIR
PLS5	SSR	2	TAIR
nga361	SSR	2	TAIR
t32f646516	SSR	2	(O'Neill *et al.*, 2008)[1)]
BIO2	SSR	2	TAIR
nga172	SSR	3	TAIR
nga126	SSR	3	TAIR
nga162	SSR	3	TAIR
ciw11	SSR	3	TAIR
F1P2-TGF	SSR	3	TAIR
ciw27	SSR	3	TAIR
nga6	SSR	3	TAIR
nga8	SSR	4	TAIR
nga1111	SSR	4	TAIR
DET1.2	SSR	4	TAIR
ciw6	SSR	4	TAIR
ciw7	SSR	4	TAIR
nga1139	SSR	4	TAIR
nga1107	SSR	4	TAIR
ciw15	SSR	5	TAIR
nga249	SSR	5	TAIR
CA72	SSR	5	TAIR
nga151	SSR	5	TAIR
nga139	SSR	5	TAIR
SO262	SSR	5	TAIR
SO191	SSR	5	TAIR
nga129	SSR	5	TAIR
ciw10	SSR	5	TAIR
MBK5	SSR	5	TAIR

Marker Name	Marker Type	Chr.	Source
EH1-1	SCAR	1	(Häffner *et al.*, 2010)[2)]
EH1-2	SCAR	1	Häffner *et al.* 2010
EH1-3	SCAR	1	Häffner *et al.* 2010
EH2-1	SCAR	2	Häffner *et al.* 2010
EH2-3a	SCAR	2	Häffner *et al.* 2010
EH2-4	SCAR	2	Häffner *et al.* 2010
EH2-6	SCAR	2	See Table IIA
EH2-7	SCAR	2	See Table IIA
EH2-10	SCAR	2	See Table IIA
EH2-12	SCAR	2	See Table IIA
EH3-1	SCAR	3	Häffner *et al.* 2010
EH3-3	SCAR	3	Häffner *et al.* 2010
EH3-5	SCAR	3	See Table S2
EH4-1	SCAR	4	Häffner *et al.* 2010
EH4-2	SCAR	4	See Table IIA
EH5-4	SCAR	5	See Table IIA
EH5-5	SCAR	5	See Table IIA
EH5-7	SCAR	5	Häffner *et al.* 2010
EH5-8	SCAR	5	See Table IIA
EH5-9	SCAR	5	Häffner *et al.* 2010
EH5-10	SCAR	5	See Table IIA
BLC1-5	CAPS	1	See Table IIB
BLC1-6	CAPS	1	See Table IIB
BLC1-21	CAPS	1	See Table IIB
BLC1-26	CAPS	1	See Table IIB
BLC1-28	CAPS	1	See Table IIB
BLC1-34	CAPS	1	See Table IIB
BLC2-1	CAPS	2	See Table IIB
BLC2-2	CAPS	2	See Table IIB
BLC2-5	CAPS	2	See Table IIB
BLC2-7	CAPS	2	See Table IIB
BLC2-9	CAPS	2	See Table IIB
BLC2-17	CAPS	2	See Table IIB
BLC4-22	CAPS	4	See Table IIB

[1)] O'Neill CM, Morgan C, Kirby J, Tschoep H, Deng PX, Brennan M, Rosas U, Fraser F, Hall C, Gill S *et al*: **Six new recombinant inbred populations for the study of quantitative traits in *Arabidopsis thaliana***. *Theor Appl Genet* 2008, **116**:623-634.

[2)] Häffner E, Karlovsky P, Diederichsen E: **Genetic and environmental control of the *Verticillium* syndrome in *Arabidopsis thaliana***. *BMC Plant Biol* 2010, **10**:235.

Table II: Technical information on new markers for the (Bur×L*er*) RIL Population

A. SCAR-Markers

			Tm		T_m (°C)	T_a (°C)	t_e (s)	Fragment Bur (bp)	Fragment L*er*(bp)
EH2-6		GTGTCTCAAAAACTTGGATGTC	62	TCGAGTAGGTCAAGCCAACG	62	55	30	183	173
EH2-7		CTGTGGTCGAACAGTCGAAG	62	CATCGTCACACACAGGCGGG	66	55	30	278	269
EH2-10		CTCTTCTTCGTCATACCATCC	62	TTTCTTACAGCACTTCTACTTT	58	55	30	214	203
EH2-12		TAGTGTTGTTGGGGTCGTC	58	GGATGAGAATTTCCGGGATTTT	62	55	30	238	226
EH3-5		GTGTTGTTTCGTGCTTCCG	58	CTCGTAGTGCATCCGACTCC	64	55	30	202	193
EH4-2		AAGTGTGGGCTAGCTGATAA	58	AGCCAATAGATGGCTACTCG	60	55	30	163	173
EH5-4		GCTCGCCGTAAAGCTGAAGC	64	GATAGCTGCCGTCACCAAAGACC	72	55	30	184	195
EH5-5		CTCTGTGTTCTAGTTCCCCA	60	CCACATCACCAGGACGCC	60	55	30	282	302
EH5-8		TCACGATCTGTCTTCTCCGG	62	GGATACTGTCCAAGCAATTCCAC	68	55	30	292	351
EH5-10		CGCAGGAGTTTTCTCTCCAA	60	CCTTCGATTCCACTCTTCTC	60	55	30	228	216

B. CAPS-Markers

Marker Name	Based on Polymorphism	Forward Primer	T_m (°C)	Reverse Primer	T_m (°C)	T_a (°C)	t_e (s)	Restr. Enzyme	Fragments Bur (bp)	Fragments Ler(bp)
BLC1-5	Ossowski_33803	GACAACCTCCAGATCGGTGT	62	ATGTTCCTGTAGAACAGCCGG	49.2	55	30	BsmFI	187, 44	231
BLC1-6	Ossowski_41322	CTTCTAACTACCAATGATCTC	43.4	GTGTGCCACGCAGATGACTG	64	55	40	AluI	250	169, 81
BLC1-21	Ossowski_237400	GCACATGCAGTACACCAACC	62	ATGACAGAAATAGAGCGAGC	58	55	40	None/LP	>236	236
BLC1-26	Ossowski_242350	GCCGTTGCAGTCACTTGACTC	51.2	GGATATATGGATCCAGAGTAC	45.3	50	40	MnlI	242	126, 116
BLC1-28	Ossowski_1182884	GGTCTTGGATTAGCTTAGGC	60	AATAGCGAGGAGTTTCCAGT	58	55	30	NdeI	214	194,21
BLC1-34	Ossowski_126257	TACGTCTATGAGGATACGTC	58	CTCCATGTGGTCAGGCGGTGG	55	49	40	None/LP	<217	217
BLC2-1	Ossowski_391600	CTGTTGTTTCTTACGTCACTG	45.3	CTGTATGTTCCCCATGATTCTTAC	48.9	55	40	Tru1I	345, 57	402
BLC2-2	Ossowski_1194744	GGCTGTCCATTTGCATCTTAC	47.3	CTCACGAAAAGTCCATGTTGTCTAC	50.9	55	40	BseRI	208, 89	297
BLC2-5	BKN000004572	GCATAGAAGCGATATGAGAATA	44.1	CATCTACTGGATTCCGGCTGG	51.2	55	40	AflIII	400, 83	483
BLC2-7	Ossowski_393125	GCTCTTTACTCCGATCATCG	60	GAGGTATATCGTGAACCAGTTT	62	55	40	HincII	179, 106	286
BLC2-9	Ossowski_400251	AGTCATTAAGTTAGGTAATTT	37.5	TGGTAGTTTATGTACAAGCTA	41.4	50	40	None/LP	265	>265
BLC2-17	Ossowski_403057	GAGTAGTTAACTCTATCACGC	45.3	TACTTAATTGAGACAAGAGG	54	55	40	AluI	229	183, 45
BLC4-22	Ossowski_780572	CAATCCCTTGAAGTTCTGAAAC	46	CTGTGGCACCGGATTCAAG	60	55	40	TaqI	298	196, 102

Abbreviations: T_m = melting temperature, T_a = annealing temperature, t_e = elongation time, bp = base pairs, LP = length polymorphism.
