# Supplementary material for: ERECTA, salicylic acid, abscisic acid, and jasmonic acid modulate quantitative disease resistance of Arabidopsis thaliana to Verticillium longisporum
Source: BMC Plant Biol. 2014 Apr 1;14:85. doi: 10.1186/1471-2229-14-85 (PMC4021371; doi:10.1186/1471-2229-14-85)
Supplement: Additional file 2 — Genetic and physical map. Shows the genetic map and the physical map of the (Bur×Ler) RIL population. The genetic map shows marker distances for all chromosomes in cM, the physical map shows the location of markers in kilobases (kb) according to the AGI map [48]. [file 1471-2229-14-85-S2.pdf]

Additional File 2: Genetic and physical map

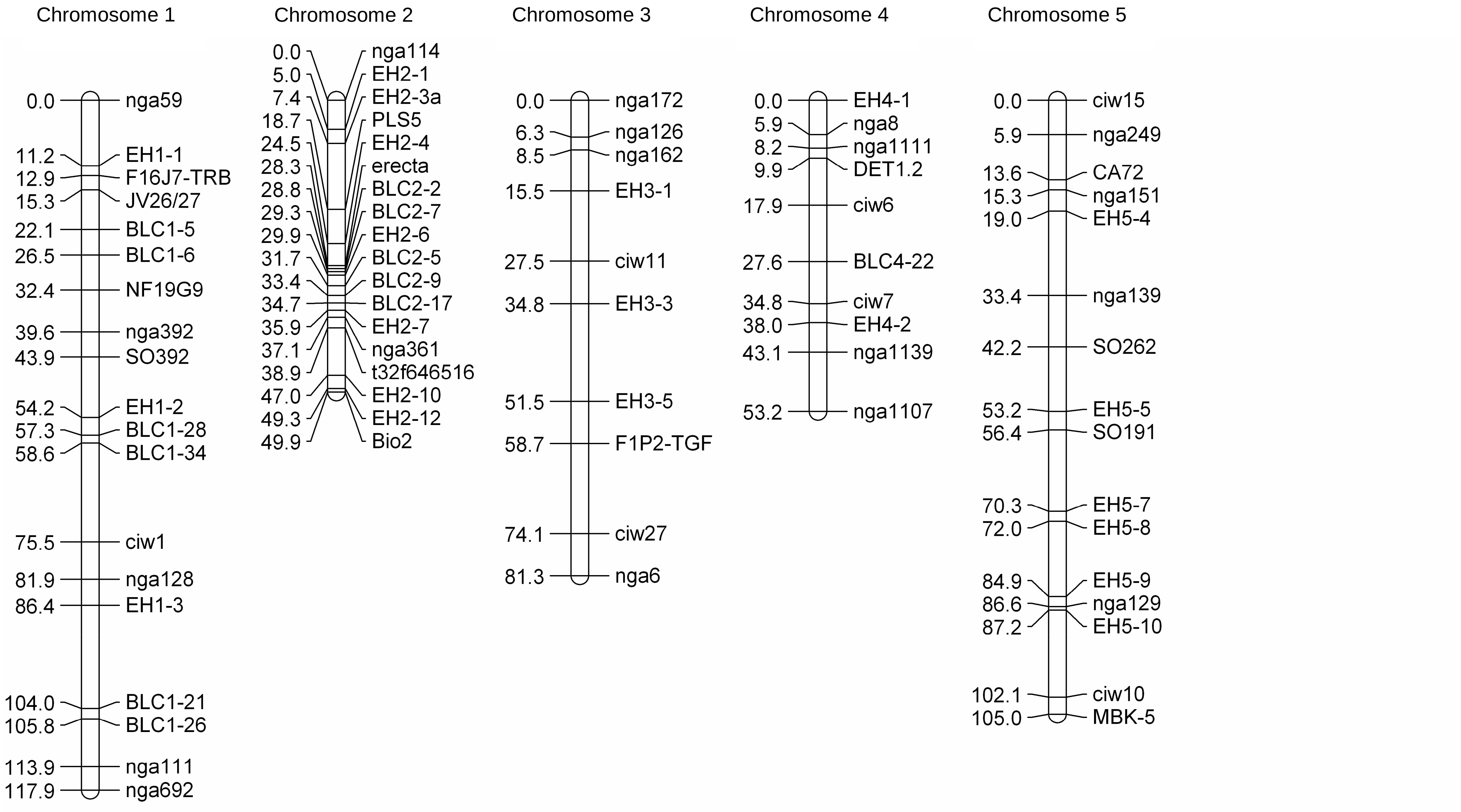

Figure I: Genetic map for the Bur×Ler Recombinant Inbred Line population created using the Haldane mapping function. Marker names are provided on the right side of each chromosome. Numbers on the left side stand for centiMorgan (cM)

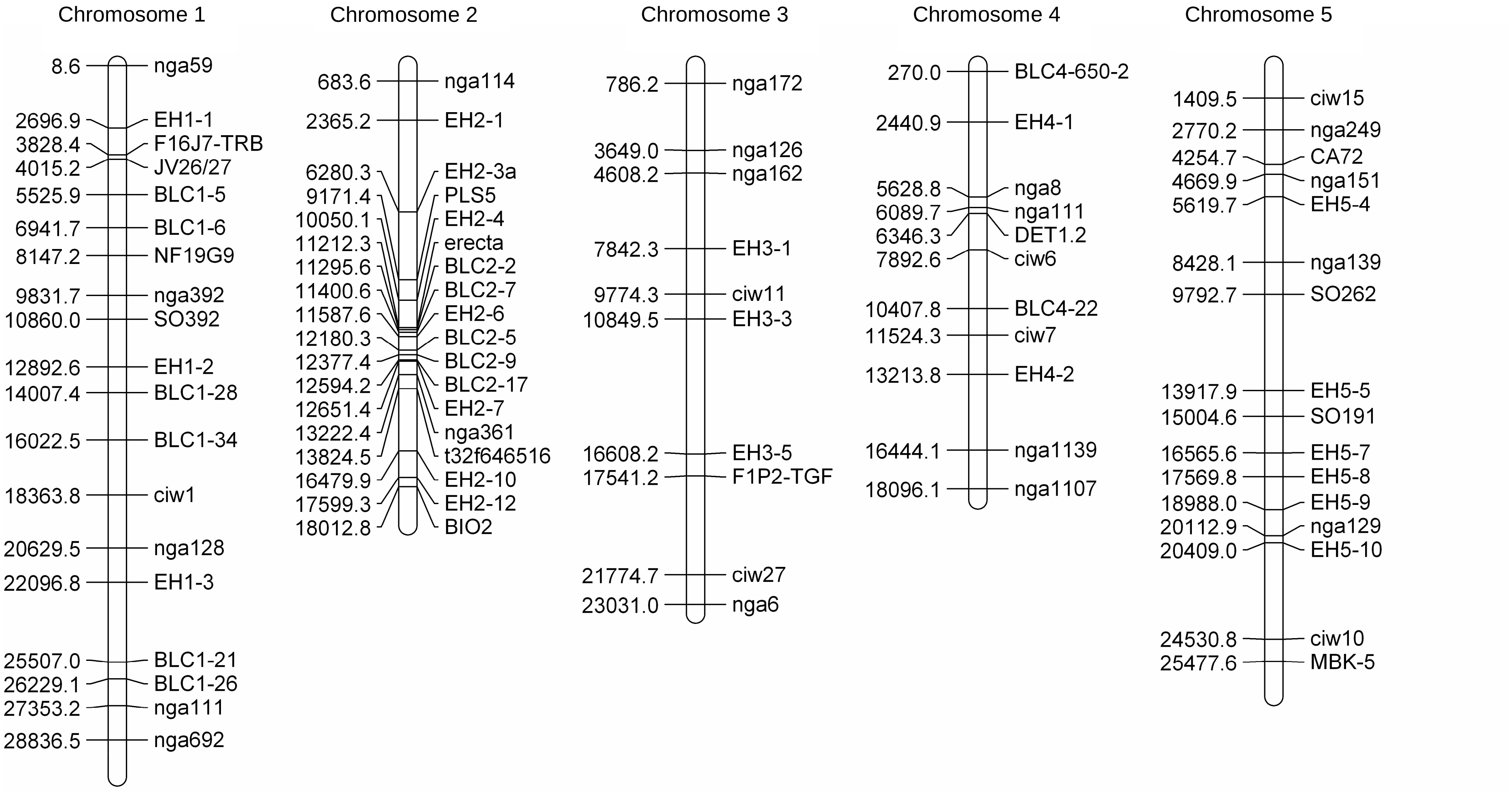

Figure II: Physical map for the (Bur×L*er*) RIL population according to AGI (The Arabidopsis Genome Initiative, 2000. Analysis of the genome sequence of the flowering plant *Arabidopsis thaliana*. Nature 408, 796-815). Marker names are provided on the right side of each chromosome. Numbers on the left side stand for kilobases (kb).
