# Supplementary material for: ERECTA, salicylic acid, abscisic acid, and jasmonic acid modulate quantitative disease resistance of Arabidopsis thaliana to Verticillium longisporum
Source: BMC Plant Biol. 2014 Apr 1;14:85. doi: 10.1186/1471-2229-14-85 (PMC4021371; doi:10.1186/1471-2229-14-85)
Supplement: Additional file 3 — QTL information. Lists information for all QTL detected in the present study with 94 (Bur×Ler) RILs in two separate experiments. QTL for the traits systemic colonization, stunting resistance and resistance to chlorosis detected with different parameters are listed in a table. The chromosomal location, the LRS peak position(s), the LOD score, the additive component, the explained trait variance, and allelic means are given for each QTL. [file 1471-2229-14-85-S3.pdf]

Additional File 3: QTL information

Overview of QTL for *V. longisporum*-related traits detected in a QTL mapping study with 94 (Bur×L*er*) RILs in two separate experiments. Most traits were assessed using different parameters. QTL are listed separately for each parameter and experiment. QTL for a trait detected with different parameters or in different experiments were given the same name when the confidence intervals were overlapping. See Figure 2 for visualisation of QTL and confidence intervals.

**Trait** – Parameter	Exp.	Chr.	QTL name	Peak pos. (cM)	Nearest marker	LRS	LOD	Add.	Var. (%)	Mean±SD for RILs with alleles from	
										Bur	L*er*
**Systemic Colonization** – % colonized shoot segments	1	2	*vec1*	22/23	EH2-4	8.1*	1.76	9.30/9.20	8	22.52±24.79	39.14±33.00
				29	BLC2-2/BLC2-7	12.5**	2.72	10.71	12	29.10±9.32	41.53±32.37
				30	EH2-6	12.8**	2.78	10.76	13	19.92±21.98	40.85±32.50
				33	BLC2-5/BLC2-9	10.4*	2.26	9.97	10	21.41±23.68	40.15±31.90
				36	EH2-7	11.5*	2.5	10.22	12	20.89±24.43	39.71±31.13
				39	t32f646516	15.7**	3.41	11.83	15	18.86±22.83	39.94±31.27
		4	*vec2*	7	nga8/nga1111	15.0**	3.26	11.76	15	19.45±25.57	42.02±29.63
				9	nga1111/DET1.2	14.3**	3.11	11.55	14	18.20±25.28	41.26±29.41
				13/14	DET1.2/ciw6	14.9**	3.24	12.63/12.66	15	17.14±24.81	39.47±29.78
			vec3	22/23	ciw6	14.4**	3.13	12.70/12.74	14	18.08±26.26	40.21±28.94
				37	ciw7/EH4-2	7.6*	1.65	8.73	8	23.31±28.73	37.02±29.60
	2	2	*vec1*	23	EH2-4	13.8**	3.0	12.03	14	37.47±28.96	59.29±27.53
				28	erecta	19.3***	4.19	13.19	19	35.74±28.1	62.17±25.62
				30	EH2-6	18.2***	3.96	12.86	18	35.74±27.43	60.67±27.64
				33	BLC2-5/BLC2-9	9.5*	2.07	9.72	10	39.38±29.11	57.59±28.31
				36	EH2-7	10.4**	2.26	9.92	11	38.11±29.94	56.96±26.85
				39	t32f646516	11.1**	2.41	10.24	11	37.60±29.92	56.80±26.86
		4	vec2	6	nga8	13.7**	2.98	11.18	14	35.79±28.95	58.17±27.26
				13	DET1.2	9.3*	2.02	10.24	10	35.88±30.39	54.39±27.67
			vec3	25	BLC4-22	11.7**	2.54	11.48	12	36.51±30.21	56.80±26.64
				34	ciw7	15.9**	3.46	12.38	16	36.01±29.47	58.31±29.47
				37	ciw7/EH4-2	17.3***	4.12	13.04	17	36.01±29.47	58.31±29.47

**Systemic Colonization** – pg *Verticillium* DNA/mg Fresh Weight	1	2	*vec1*	22	PLS5/EH2-4	11.5**	2.74	36.49	12	34.93±66.23	98.37±118.08
				26	EH2-4/erecta	11.7**	2.54	36.19	12	33.14±64.75	99.71±118.62
				29	BLC2-2	14.9**	3.24	38.39	15	25.81±43.44	101.92±119.28
				30	EH2-6	14.9**	3.24	38.39	15	24.29±39.46	99.02±117.14
				33	BLC2-5/BLC2-9	10.7*	2.33	33.60	11	30.78±52.08	93.59±115.38
				36	EH2-7	10.8*	2.35	32.87	11	31.54±54.05	91.75±117.26
				39	t32f646516	12.0**	2.61	34.72	12	31.01±55.95	91.28±112.88
		4	*vec2*	6	nga8	9.3*	2.02	30.53	10	32.93±79.56	94.72±116.29
	2	*2*	*vec1*	24	EH2-4	7.8*	1.70	25.38	8	37.59±49.94	86.07±105.48
				29	BLC2-2	10.5*	2.28	28.56	11	35.60±27.93	92.45±105.79
				30	EH2-6	10.9*	2.37	28.88	11	34.03±46.59	90.23±103.7
				33	BLC2-5/BLC2-9	6.8*	1.48	23.56	7	39.18±59.58	84.16±100.51
				35	BLC2-17	7.5*	1.63	24.23	8	39.09±60.36	91.10±103.70
				39	t32f646516	7.1*	1.54	23.49	8	36.91±60.54	88.98±100.50
**Stunting Resistance** – Performance Height (%)	1	1	*stre2*	103	BLC1-21	22.4***	4.87	10.63	19	46.66±21.35	64.69±20.44
				106/107	BLC1-26	22.9***	4.98	10.70	21	45.75±21.33	67.34±18.30
			stre1	117	nga692	16.8**	3.65	10.11	18	44.32±22.53	63.99±20.03
		2	*r-stre1*	27	erecta	35.6***	7.74	-13.10	32	66.28±17.64	40.85±19.86
				29	BLC2-7	32.8***	7.13	-10.35	29	65.84±18.01	41.00±20.16
			r-stre2	40/41	t32f646516	21.5***	4.67	-10.69	20	64.64±20.32	44.53±20.40
		4	*r-stre3*	21	ciw6	12.7**	3.02	8.85	13	47.50±21.34	61.99±20.72
	2	2	*r-stre1*	28	erecta	15.0**	3.26	-7.47	15	90.75±21.69	75.79±13.12
				29	BLC2-7	14.4**	3.13	-7.38	14	90.56±21.89	75.72±13.28
			r-stre2	42	t32f646516	7.3*	1.59	-5.85	7	88.30±24.52	78.45±12.20
**Chlorosis** – No. of chlorotic leaves in inoculated plants	1	4	*r-chl1*	6	nga8	27.6***	6.00	-0.61	26	4.34±1.04	3.11±1.07
				10	DET1.2	29.3***	6.37	-0.64	27	4.42±1.06	3.14 ±1.03
**Chlorosis** – % chlorotic leaves in inoculated plants	1	2	*r-chl2*	33	BLC2-5/BLC2-9	11.7**	2.54	-3.46	13	29.68±9.48	23.29±7.81
		4	*r-chl1*	6	nga8	13**	2.83	-3.61	15	30.46±8.70	23.23±8.67
				10	DET1.2	8.9*	1.93	-3.16	10	30.30 ±7,22	23,98±9.85
**Chlorosis** – Difference in mean No. of chlorotic leaves between inoculated and mock-inoculated plants	1	1	*r-chl3*	50/51	EH1-2	11.9**	2.59	-0.44	12	2.48±1.11	1.72±1.09
				56	BLC1-28	13.3**	2.89	-0.43	13	2.55±1.03	1.74±1.07
				59/60	BLC1-34	14.8**	3.22	-0.44/-0.46	15	2.53±1.03	1.61±0.96
		4	*r-chl1*	7	nga8/nga1111	11.3**	2.46	-0.40	11	2.52±1.02	1.76±1.16
				11	DET1.2	15.7**	3.41	-0.48	16	2.63±1.03	1.72±1.08

**Development Time** – Days to flowering	1	4	*dt1*	3	EH4-1/nga8	12.2**	2.65	-3.21	13	28.76±9.84	23.21±6.16
			dt2	11/12	DET1.2	15.5**	3.37	-3.49	15	29.46±9.96	23.17±6.03
	2	4	*dt1*	2	EH4-1	15.8**	3.43	-1.48	16	24.03±3.81	21.63±2.63
			dt2	9/10	DET1.2	15.3**	3.33	-1.40	15	24.18±3.97	21.68±2.60

Asterisks denote the genome-wide confidence level for a QTL as determined by permutation test (P = 0.37, 0.95 and 0.99 respectively). Negative values for the additive component stand for Bur origin of the increasing allele, positive values for L*er* origin.

(Abbreviations: Exp. = experiment, Chr. = chromosome, Peak pos. = peak position, cM = centiMorgan, LRS = Likelihood Ratio Statistic, LOD = Likelihood of Odds, Add. = Additive component, Var. = explained trait variance).
