# Supplementary material for: ERECTA, salicylic acid, abscisic acid, and jasmonic acid modulate quantitative disease resistance of Arabidopsis thaliana to Verticillium longisporum
Source: BMC Plant Biol. 2014 Apr 1;14:85. doi: 10.1186/1471-2229-14-85 (PMC4021371; doi:10.1186/1471-2229-14-85)
Supplement: Additional file 4 — Frequency distribution of phenotypic data. Shows the frequency distribution histograms for the phenotypic data used in QTL mapping. Histograms are shown for the parameters “% colonized shoot segments”, “pg Verticillium DNA/mg fresh weight”, “performance height”, “Mean number of yellow leaves in V. longisporum-inoculated plants”, “Mean number of yellow leaves related to total rosette leaf number”, and “Mean difference in yellow leaves between inoculated and mock-inoculated plants”. [file 1471-2229-14-85-S4.pdf]

Additional File 4: Frequency distribution of phenotypic data

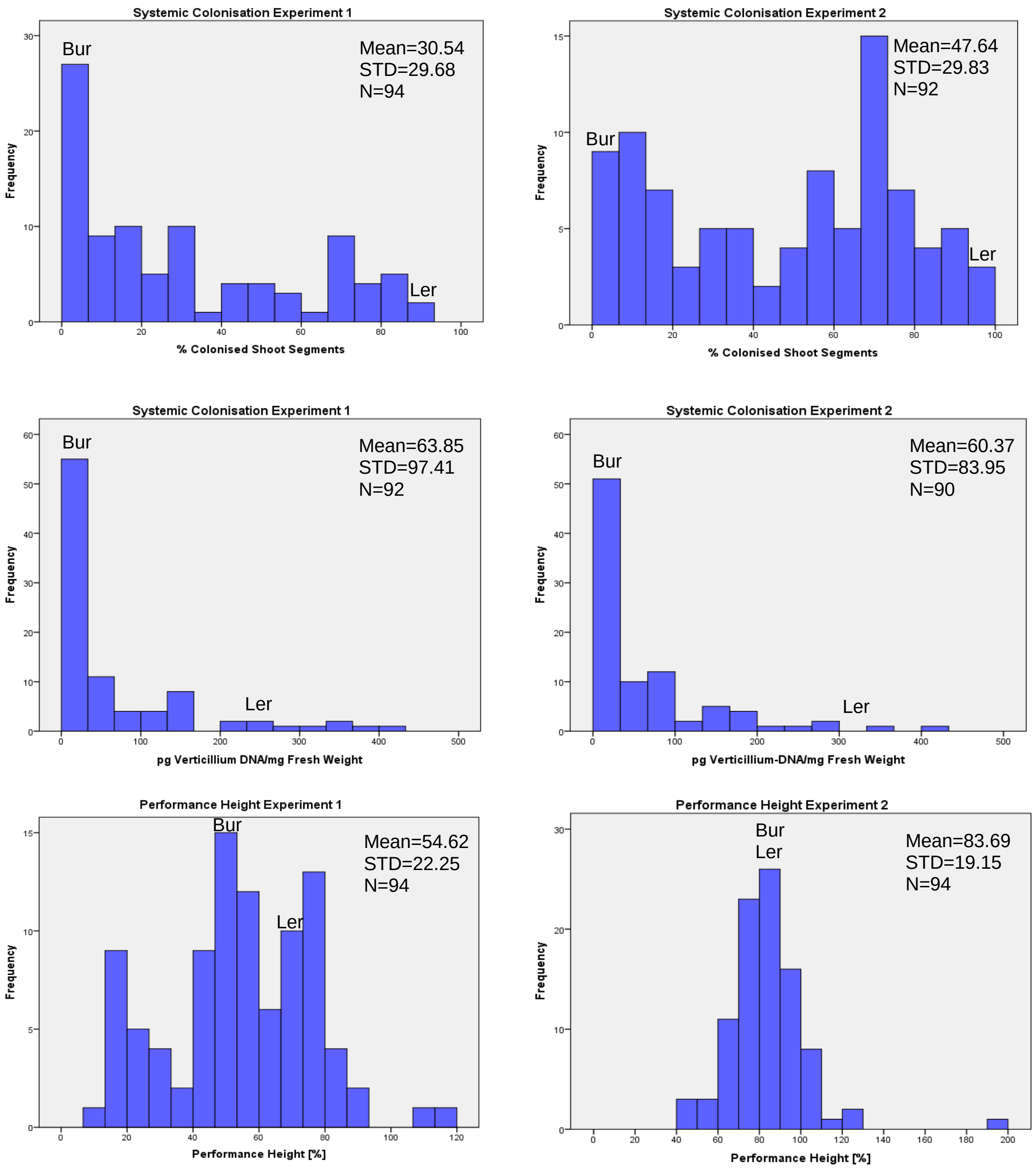

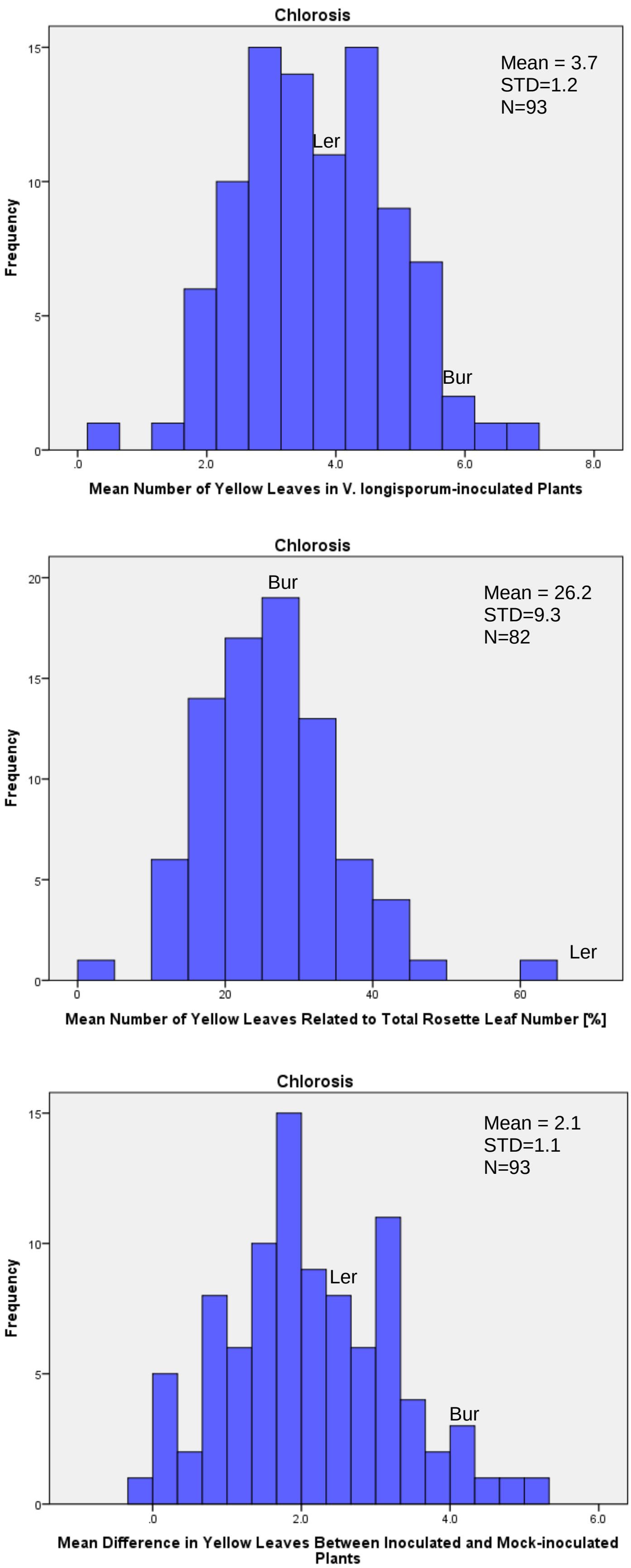

Frequency distributions for *V. longisporum*-related parameters used in QTL-mapping in the (Bur×L*er*) RIL population.
