# Supplementary material for: ERECTA, salicylic acid, abscisic acid, and jasmonic acid modulate quantitative disease resistance of Arabidopsis thaliana to Verticillium longisporum
Source: BMC Plant Biol. 2014 Apr 1;14:85. doi: 10.1186/1471-2229-14-85 (PMC4021371; doi:10.1186/1471-2229-14-85)
Supplement: Additional file 5 — Systemic colonization of Erecta signalling mutants and corresponding WT-lines. Contains a bar chart visualizing systemic colonization of erecta mutants, agb1-1 mutant and the corresponding WT genotypes. It provides evidence that Erecta is not involved in mediating resistance to systemic colonization by V. longisporum. [file 1471-2229-14-85-S5.pdf]

Additional File 5: Systemic colonization of *Erecta* signalling mutants and corresponding wildtypes

Additional File 5 provides evidence that *Erecta* is not involved in mediating resistance to systemic colonization by *V. longisporum*.

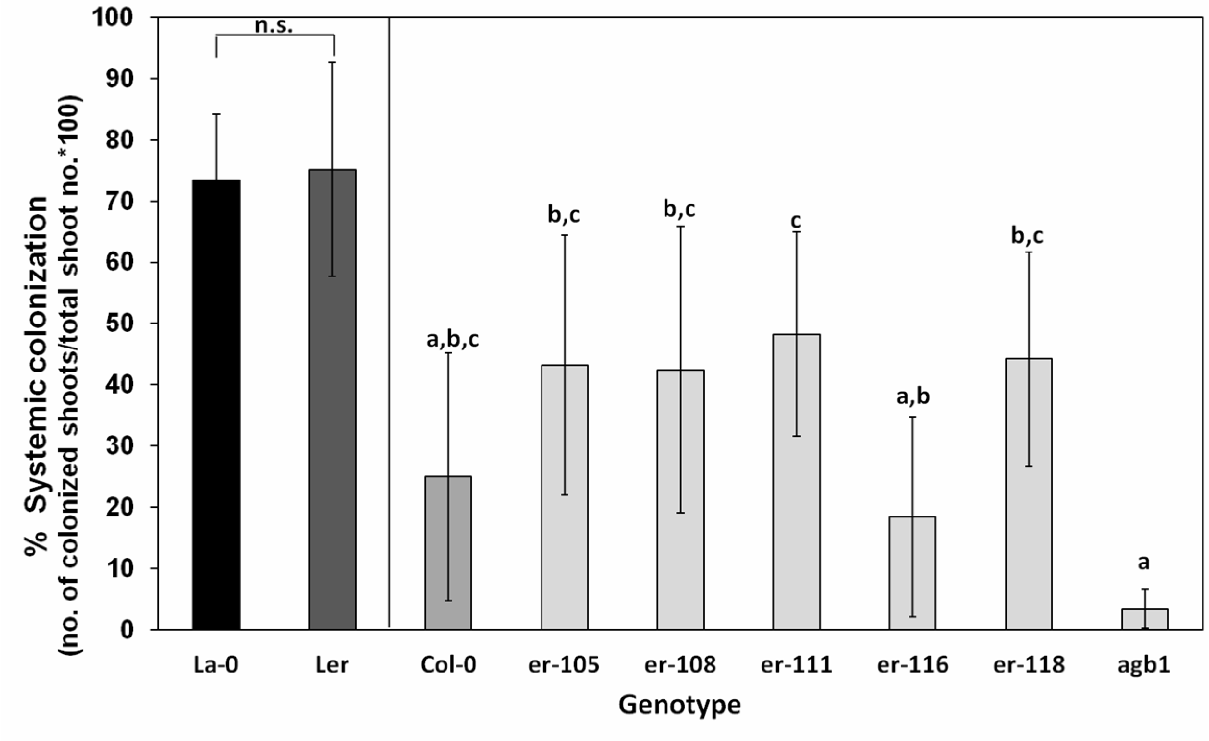

Systemic colonization by *V. longisporum* in *Erecta*-signalling mutants and corresponding WT lines. La-0 is the corresponding WT for L*er*, Col-0 for *er-105*, *er-108*, *er-116*, *er-118,* and *agb1-1*. Different WT/mutant combinations are separated by a vertical bar. Significance of La-0/L*er* -differences was tested by t-tests (N = 10); differences between lines with Col-0-background by one-way ANOVA and subsequent multiple comparisons (Tukey test, N = 10). Means marked with different letters differed significantly at $p < 0.05$. Vertical bars denote standard deviations.
