# Supplementary material for: ERECTA, salicylic acid, abscisic acid, and jasmonic acid modulate quantitative disease resistance of Arabidopsis thaliana to Verticillium longisporum
Source: BMC Plant Biol. 2014 Apr 1;14:85. doi: 10.1186/1471-2229-14-85 (PMC4021371; doi:10.1186/1471-2229-14-85)
Supplement: Additional file 6 — SA contents in Bur, Ler and NILs. Contains a bar chart visualizing SA contents in Bur, Ler and two NILs differing in the vec1 region. The data provide evidence that SA hyperinduction, which is characteristic for Bur, is not associated with the vec1 locus. [file 1471-2229-14-85-S6.pdf]

Additional File 6: SA contents in Bur, L*er* and NILs

Additional File 6 provides evidence that SA hyperinduction, which is characteristic for Bur, is not associated with the *vec1*-locus.

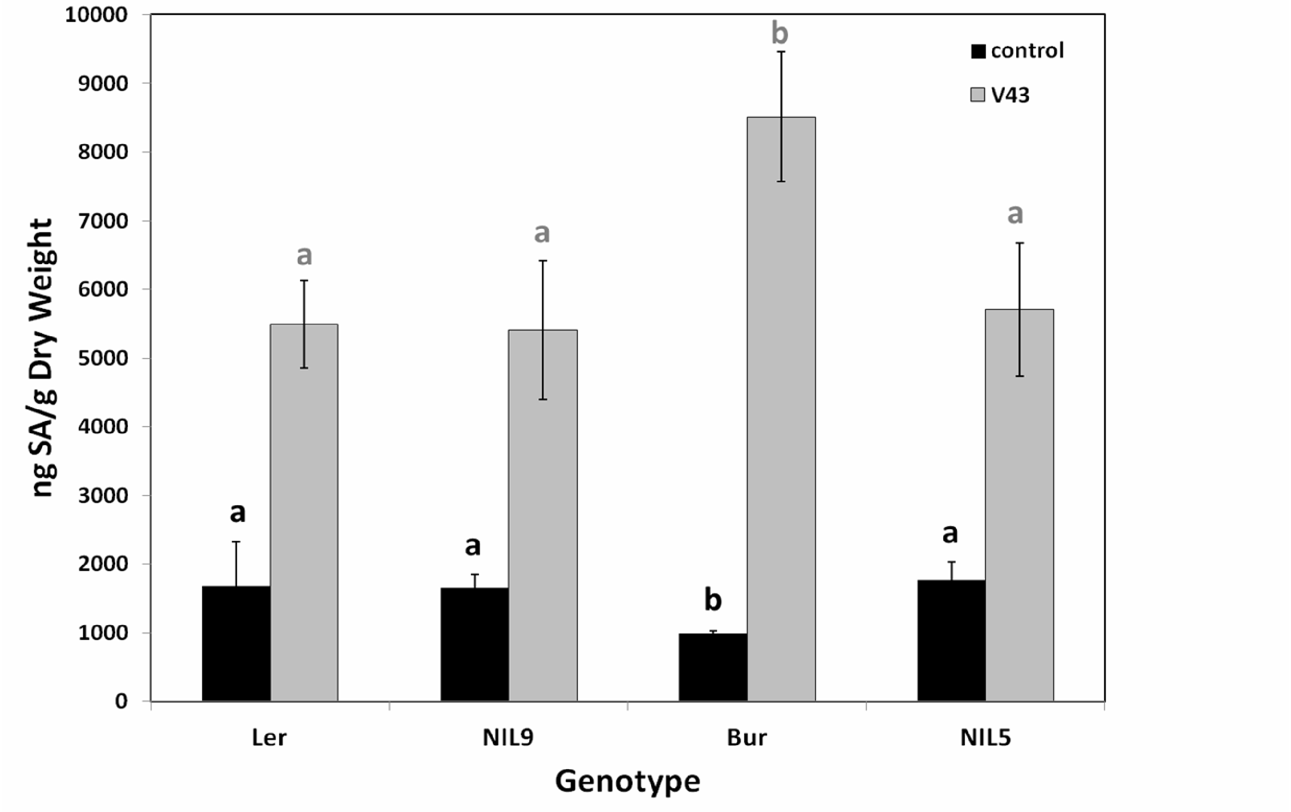

SA-content of Bur, L*er* and two near-isogenic lines: NIL9 (L*er* -alleles in the variable region) and NIL5 (Bur -alleles). Differences between mock- and *V. longisporum*-inoculated plants were significant (n = 6-7, $p < 0.05$). Means of plants marked with different letters **within one treatment** (mock- and *V. longisporum*-inoculated, respectively) differed significantly at $p < 0.05$. (one-way ANOVA and post-hoc Tukey test). Vertical bars denote standard deviations.
