# Supplementary material for: ERECTA, salicylic acid, abscisic acid, and jasmonic acid modulate quantitative disease resistance of Arabidopsis thaliana to Verticillium longisporum
Source: BMC Plant Biol. 2014 Apr 1;14:85. doi: 10.1186/1471-2229-14-85 (PMC4021371; doi:10.1186/1471-2229-14-85)
Supplement: Additional file 7 — Phytohormone contents of erecta mutants, corresponding WT-lines and Bur. Contains three bar charts visualizing SA, ABA and JA contents of Erecta signalling mutants and corresponding WT lines after mock-inoculation and V. longisporum-infection. Additional file 7 provides evidence that differences in phytohormone response to V. longisporum between Bur and Ler are not caused by Erecta. [file 1471-2229-14-85-S7.pdf]

Additional File 7: Phytohormone contents of *erecta* mutants, corresponding WT-lines and Bur

Additional File 7 provides evidence that differences in phytohormone response to *V. longisporum* between Bur and L*er* are not caused by *Erecta*.

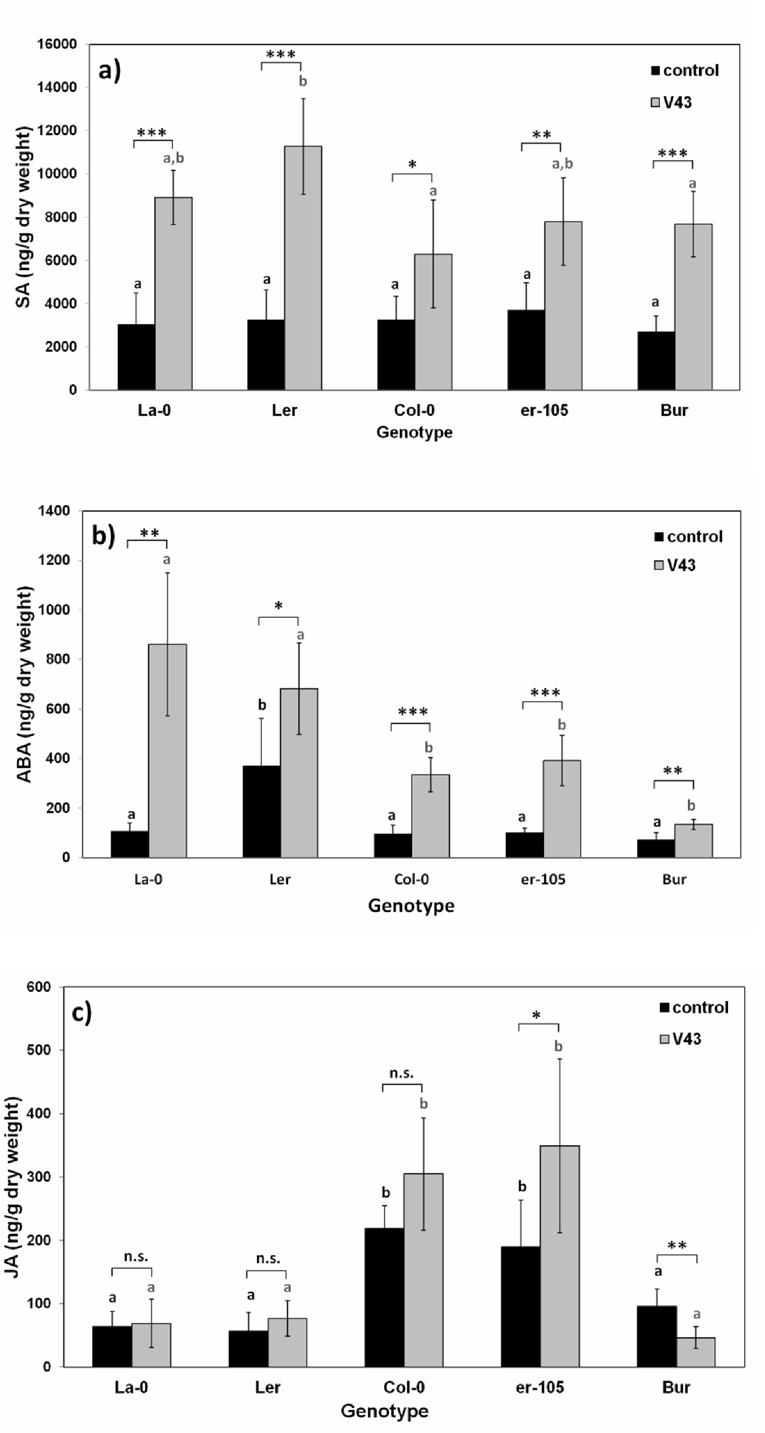

Contents of the phytohormones a) SA, b) ABA and c) JA in *erecta* mutants and their corresponding WT-lines, and Bur upon infection. Asterisks refer to the significance level of differences between mock-inoculated and *V. longisporum*-treated plants **within one genotype** (t-test, n = 6). **Within one treatment**, genotype means were compared by one-way ANOVA and multiple comparisons (Tukey test). Means marked with different letters differed significantly at $p < 0.05$. Vertical bars denote standard deviations.
